# Antiparasitic Activity of Sesquiterpene γ-Lactones and the Search for New Pharmaceutical Agents

**DOI:** 10.3390/ijms27177971

**Published:** 2026-09-07

**Authors:** Sergazy M. Adekenov, Laura C. Laurella, Shynggys Sergazy, Rachel Napoles Rodriguez, Augusto E. Bivona, Juan M. Viecenz, Orlando G. Elso, Aldana M. Corlatti, Nadia T. Mirakian, Esteban Bontempi, Paola A. Barroso, Dmitriy L. Savchenko, Balzhan Z. Medeubaeva, Gulimzhan S. Adekenova, Artur T. Boldysh, Anar N. Zhabayeva, Valeria P. Sülsen

**Affiliations:** 1 JSC “Research Production Center “Phytochemistry”, 100009 Karaganda, Kazakhstan; shynggys.sergazy@gmail.com (S.S.); savchenkod_pharmatech@mail.ru (D.L.S.); medeubayeva_bz@mail.ru (B.Z.M.); g.uuuuu@bk.ru (G.S.A.); art.boldysh@gmail.com (A.T.B.); a.zhabaeva@phyto.kz (A.N.Z.); 2 CONICET-Universidad de Buenos Aires, Instituto de Química y Metabolismo del Fármaco (IQUIMEFA), Buenos Aires C1113AAD, Argentina; c.laurella@docente.ffyb.uba.ar (L.C.L.); orlando.elso@gmail.com (O.G.E.); aldanamalencorlatti@gmail.com (A.M.C.); nadiatmirakian@gmail.com (N.T.M.); 3 Universidad de Buenos Aires, Facultad de Farmacia y Bioquímica, Cátedra de Farmacognosia, Buenos Aires C1113AAD, Argentina; rnapolesrodriguez@gmail.com; 4 Universidad de Buenos Aires, Facultad de Farmacia y Bioquímica, Cátedra de Inmunología, Buenos Aires C1113AAD, Argentina; abivona@docente.ffyb.uba.ar; 5 CONICET-Universidad de Buenos Aires, Instituto de Estudios de la Inmunidad Humoral (IDEHU), Buenos Aires C1113AAD, Argentina; 6 Instituto Nacional de Parasitología, ANLIS “Carlos G. Malbrán”, Buenos Aires C1282AFF, Argentina; jmviecenz@hotmail.com.ar (J.M.V.); ejbon100@gmail.com (E.B.); 7 CONICET-Universidad Nacional de Salta, Instituto de Patología Experimental “Dr. Miguel Ángel Basombrío” (IPE), Salta A4408FVY, Argentina; barrosopaola75@gmail.com

**Keywords:** sesquiterpene lactones, antiparasitic activity, *Trypanosoma brucei brucei*, *Trypanosoma cruzi*, *Leishmania amazonensis*

## Abstract

This study presents a comparative in vitro evaluation of 24 natural sesquiterpene γ-lactones and selected semisynthetic derivatives against three trypanosomatid parasites responsible for neglected tropical diseases: *Trypanosoma brucei brucei*, *Trypanosoma cruzi*, and *Leishmania amazonensis*. The compounds were screened for antiparasitic activity across different parasite life stages, and their cytotoxicity was assessed in mammalian cells. Several compounds demonstrated high inhibitory activity (>95%) at 10 µg/mL, including estafiatin (**1**), grossheimin (**4**), cynaropicrin (**5**), argolide (**2**) derivatives, and arglabin (**23**). However, a marked decrease in activity was observed at lower concentrations and in intracellular parasite models, particularly for *T. cruzi* and *L. amazonensis* amastigotes. Among the tested compounds, dihydroargolide (**3**) and pulchellin C (**19**) exhibited comparatively lower cytotoxicity and were further evaluated, yielding IC_50_ values of 10.51 ± 1.72 µg/mL and 2.51 ± 1.13 µg/mL against *T. b. brucei*, respectively. Against *L. amazonensis* promastigotes, estafiatin (**1**), 8α-chloroacetoxygrossheimin (**12**), and arglabin (**23**) showed IC_50_ values of 0.16 ± 0.13, 0.35 ± 0.18, and 0.58 ± 0.11 µg/mL, respectively, although their selectivity indices remained limited. Comparative analysis of the dataset indicates that specific structural elements, such as the α-methylene-γ-lactone moiety and selected substituents (e.g., epoxy and acetyl groups), may influence antiparasitic activity in a species-dependent manner. At the same time, the generally low selectivity indices and reduced activity in intracellular models highlight important limitations of these compounds as direct drug leads. Overall, this work provides a systematic comparative dataset and identifies preliminary structure–activity trends that may guide further chemical optimization of sesquiterpene γ-lactones as antiparasitic hit compounds.

## 1. Introduction

Human African trypanosomiasis (HAT), Chagas disease, and leishmaniasis are classified as neglected tropical diseases (NTDs) by the World Health Organization (WHO). Caused by *Trypanosoma brucei brucei*, *Trypanosoma cruzi*, and *Leishmania amazonensis*, respectively, these infections are associated with high morbidity and mortality, stigma, and social exclusion. NTDs primarily affect vulnerable populations in rural areas, conflict zones, and regions with limited access to clean water, sanitation, and health care. Poverty, social inequities, and climate change further contribute to their persistence and expansion [1].

HAT, or sleeping sickness, is transmitted to humans through the bite of infected tsetse flies. HAT occurs in two forms depending on the parasite subspecies: *T. b. gambiense*, responsible for chronic disease accounting for most cases in West and Central Africa, and *T. b. rhodesiense*, which causes an acute illness in Eastern and Southern Africa. Infection initially involves the blood and lymphatic system, producing fever, swollen lymph nodes, and malaise, before parasites invade the central nervous system, resulting in neurological symptoms such as sleep disturbances, cognitive changes, and motor impairment. Without treatment, HAT is usually fatal [2].

Chagas disease affects over seven million people worldwide, primarily in Latin America. Transmission occurs mainly through triatomine insects, but can also happen via contaminated food, congenital infection, blood transfusions, organ transplantation, or laboratory accidents. The disease progresses through an acute phase, often mild or asymptomatic, and a chronic phase, which may lead to cardiac, digestive, or neurological complications [3].

Leishmaniasis is caused by more than 20 *Leishmania* species and transmitted by infected female sandflies. The disease manifests in three main forms: visceral, the most severe and, often, fatal if untreated; cutaneous, the most common, which produces skin ulcers and permanent scars; and mucocutaneous, which affects the mucous membranes of the nose, mouth, and throat. Globally, 700,000 to one million new cases are estimated each year, with endemic regions in the Americas, Africa, the Middle East, Central Asia, and South Asia. Although leishmaniasis is treatable, early diagnosis and appropriate therapy are crucial to reduce morbidity, mortality, and long-term complications [4,5].

Currently, the arsenal of available antiparasitic drugs is limited, and many synthetic agents have been discontinued due to their high toxicity. At present, treatment of parasitic diseases relies on a restricted group of compounds: benznidazole and nifurtimox for Chagas disease [3,6]; pentamidine, eflornithine, nifurtimox, and fexinidazole for gambiense-HAT; suramin and melarsoprol for rhodesiense-HAT [2,7]; and, miltefosine, paromomycin and liposomal amphotericin B for leishmaniasis [8]. However, these drugs often affect not only the parasites but also the host. In some cases, adverse effects are not clinically evident but can accumulate over time, leading to cumulative toxicity.

The limited number of available antiparasitic drugs, together with their efficacy and safety limitations, highlights the need for new therapeutic candidates. Natural products represent an important source of structurally diverse bioactive molecules and have historically played a major role in drug discovery, with more than half of approved drugs being related to natural products or their derivatives [9]. Plant secondary metabolites encompass a broad range of chemical scaffolds, making them valuable starting points for the identification of new antitrypanosomal and antileishmanial compounds [10,11].

In this regard, plant-derived terpenoids, including sesquiterpene lactones (STLs), represent a biologically active class of natural compounds with reported antiparasitic properties. However, their pharmacological potential should be considered with caution, as many STLs contain electrophilic structural motifs that may also contribute to cytotoxic effects. Their antiparasitic activity has been associated with interactions with key enzymatic systems, including thioredoxin–glutathione reductase and glutathione-S-transferase [12]. In parasites of the genus Leishmania, both the glutathione system and the trypanothione/trypanothione reductase system play essential roles in defense against reactive nitrogen species and may contribute to drug-resistance mechanisms [13]. In *T. b. brucei* and *L. amazonensis*, trypanothione reductase is involved in maintaining redox balance and has been proposed as a potential therapeutic target [13,14]. In addition, the antiparasitic activity of sesquiterpene lactones has been linked to multiple biological effects, including modulation of host immune responses, interference with parasite metabolic processes, and disruption of parasite viability; however, these mechanisms remain incompletely understood [15,16].

Although several STLs have previously been reported to display antiparasitic activity, comparative datasets across multiple trypanosomatid species using structurally related natural products and semisynthetic derivatives remain limited. In particular, the extent to which specific structural modifications alter activity patterns across *Trypanosoma* and *Leishmania* models has not been systematically assessed within a single experimental framework. Therefore, the present study was designed as a comparative early-stage screening investigation to evaluate a panel of 24 sesquiterpene γ-lactones and semisynthetic derivatives across three trypanosomatid species (*T. b. brucei*, *T. cruzi*, and *L. amazonensis*), to perform a side-by-side analysis of natural compounds and their semisynthetic counterparts, and to identify preliminary structure–activity trends associated with selected chemical modifications.

## 2. Results and Discussion

**Figure 1:**
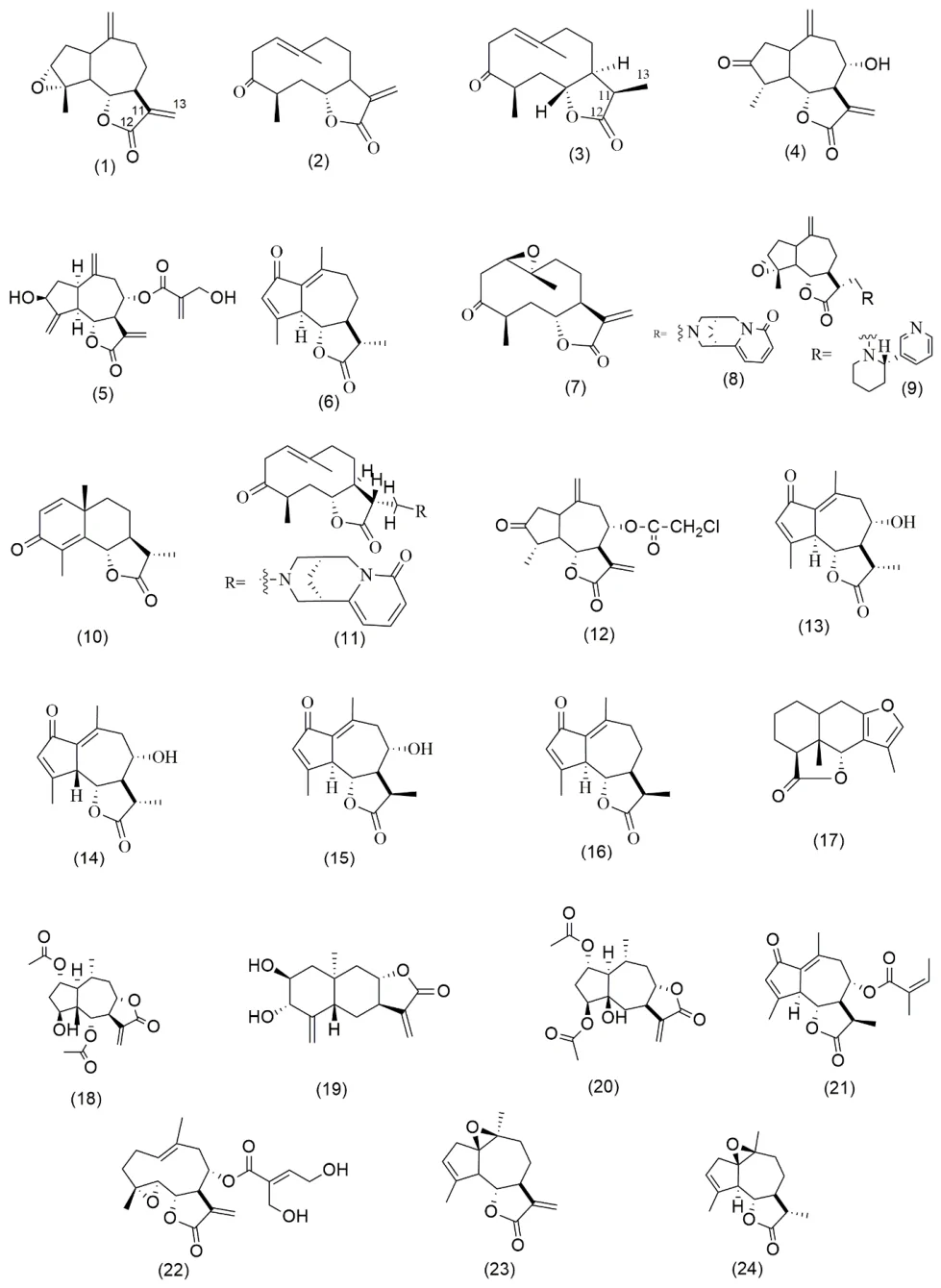
Chemical structures of the natural sesquiterpene lactones and derivatives. Estafiatin (**1**) (also known as estafietin), argolide (**2**), dihydroargolide (**3**), grossheimin (**4**), cynaropicrin (**5**), leucomisin (**6**), epoxyargolide (**7**), cytisinyl-estafiatin (**8**), anabasinyl-estafiatin (**9**), α- santonin (**10**), 13α-cytisinylargolide (**11**), chloroacetoxygrossheimin (**12**), austricin (**13**), 5β(H)-austricin (**14**), grossmisin (**15**), achillin (**16**), furanoeremophilan-14β,6α-olide (**17**), britanin (**18**), pulchellin C (**19**), inuchinenolide C (**20**), badkhyzin (**21**), stizolicin (**22**), arglabin (**23**), and arborescin (**24**).

The chemical structures of the STLs and their semisynthetic derivatives evaluated in this study are shown in Figure 1.

### 2.1. In Vitro Trypanocidal Activity Against Trypomastigotes of Trypanosoma brucei brucei

The antiparasitic activity of the STLs and their derivatives was evaluated against trypomastigotes of *T. b. brucei* (Table 1).

As shown in Table 1, the guaianolides estafiatin (**1**) and anabasinyl-estafiatin (**9**) inhibited *T. b. brucei* trypomastigotes by more than 99% at 10 µg/mL. At 1 µg/mL, estafiatin (**1**) also exhibited significant inhibition (72.91%), which was 13.6-fold higher than that of its anabasinyl derivative (**9**) at the same concentration, whereas the cytisinyl derivative (**8**) showed no detectable antiparasitic activity. These findings indicate that Michael-type amination of the exomethylene group (C11=C13) is associated with a reduction in antiparasitic activity against *T. b. brucei*. Although anabasine and cytisine are structurally related alkaloids, their estafiatin conjugates differed by more than 20-fold in activity at 10 µg/mL, suggesting a structure-dependent biological response.

Similarly, several other STLs—including the germacranolide argolide (**2**) and the guaianolides grossheimin (**4**), cynaropicrin (**5**), 8α-chloroacetoxygrossheimin (**12**), britanin (**18**), inuchinenolide C (**20**), and arglabin (**23**)—showed >95% inhibition of *T. b. brucei* trypomastigotes at 10 µg/mL. The consistent presence of the exomethylene group (C11=C13) in these more active compounds suggests that this structural feature may be associated with increased trypanocidal activity within this compound set. This observation is consistent with the recognized importance of the α-methylene-γ-lactone moiety as a major pharmacophoric element of many STLs. Chemical modification of this electrophilic center may alter its reactivity toward biological nucleophiles and consequently influence biological activity in a structure-dependent manner [12]. In contrast, dihydroargolide (**3**), which differs from argolide (**2**) in the hydrogenation of the C12–C13 bond, displayed only 53.42% inhibition at 10 µg/mL, representing a 1.85-fold loss of potency. Likewise, the 13α-cytisinyl derivative (**11**) showed a 20.74-fold reduction in activity relative to argolide (**2**).

Notably, the epoxy derivative of argolide (**7**) exhibited strong trypanocidal activity, inhibiting *T. b. brucei* by 99.18% at 1 µg/mL—9.17-fold higher than that of the natural compound (**2**). This result suggests that the introduction of the C1–C10 epoxy ring may be associated with enhanced antiparasitic activity. However, further mechanistic studies are required to confirm the specific role of this structural modification.

Furthermore, the 8α-chloroacetoxy derivative of grossheimin (**12**) showed markedly enhanced activity at 1 µg/mL, with an 11.21-fold increase in potency compared to the parent compound (**4**). This finding indicates a strong positive effect of acetylation at the C-8 hydroxyl group on activity against *T. b. brucei*.

**Figure 2:**
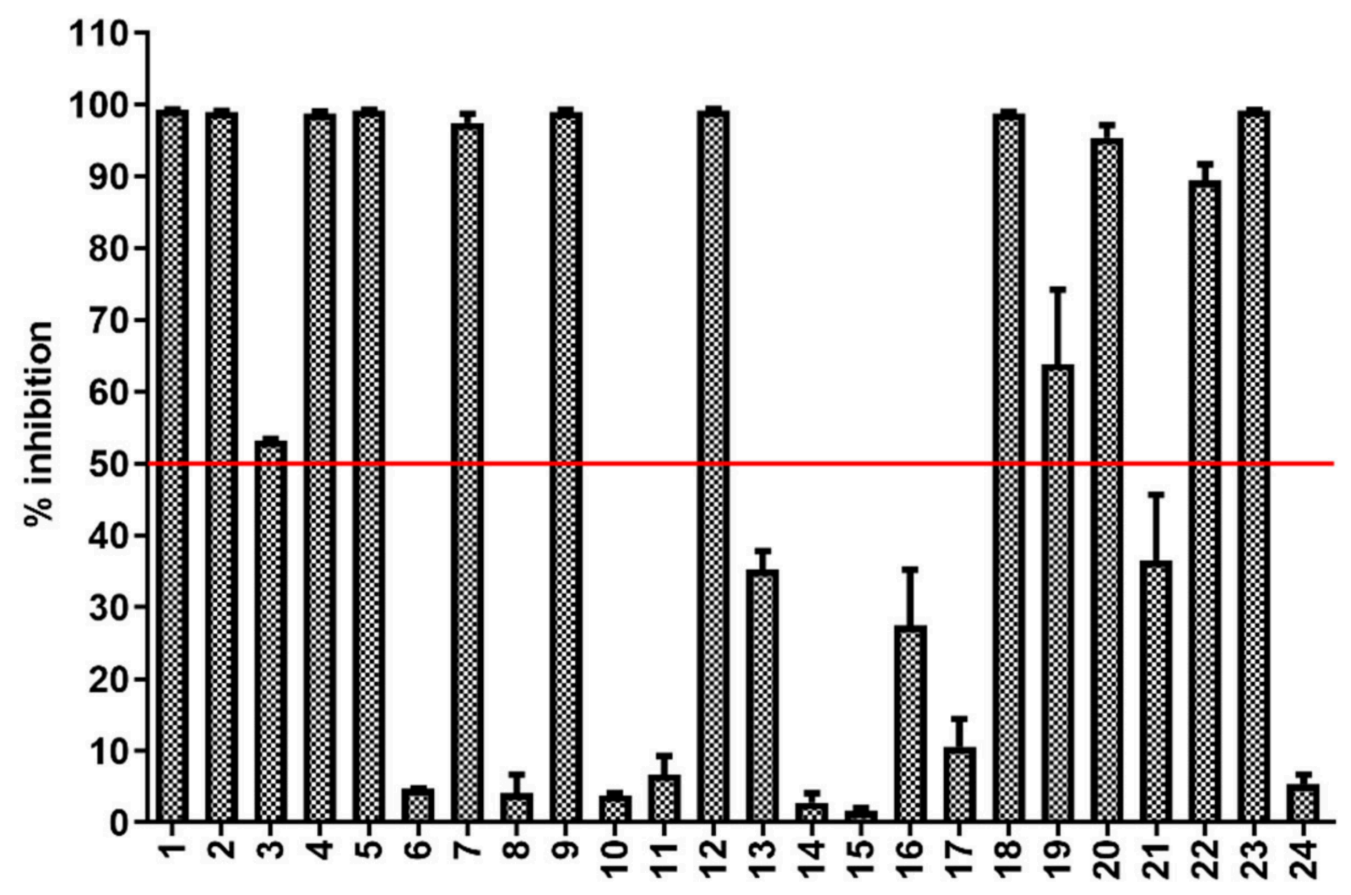
Trypanocidal activity of compounds **1**–**24** against *T. b. brucei* trypomastigotes at 10 µg/mL. Parasites were cultured for 72 h at 37 °C with 5% CO_2_ in the presence of the compounds. After the incubation period, parasites were counted using a Beckman Z2 Coulter counter. The values represent the mean ± standard deviation (SD). The red line indicates the 50% inhibition threshold.

As shown in Table 1 and Figure 2, several STLs displayed marked activity against *T. b. brucei* at 10 µg/mL. Among them, eight guaianolides—estafiatin (**1**), grossheimin (**4**), cynaropicrin (**5**), anabasinyl-estafiatin (**9**), 8α-chloroacetoxygrossheimin (**12**), britanin (**18**), inuchinenolide C (**20**), and arglabin (**23**); four germacranolides—argolide (**2**), dihydroargolide (**3**), epoxyargolide (**7**), and stizolicin (**22**); and the eudesmanolide pulchellin C (**19**) exhibited relatively high trypanocidal activity. In contrast, eight guaianolides—leucomisin (**6**), cytisinyl-estafiatin (**8**), austricin (**13**), 5β(H)-austricin (**14**), grossmisin (**15**), achillin (**16**), badkhyzin (**21**) and arborescin (**24**); the eudesmanolide—α-santonin (**10**) and the eremophilenolide furanoeremophilan-14β,6α-olide (**17**); and the germacranolide 13α-cytisinylargolide (**11**) displayed comparatively low activity under the same conditions.

### 2.2. In Vitro Activity Against Amastigotes of Trypanosoma cruzi

**Figure 3:**
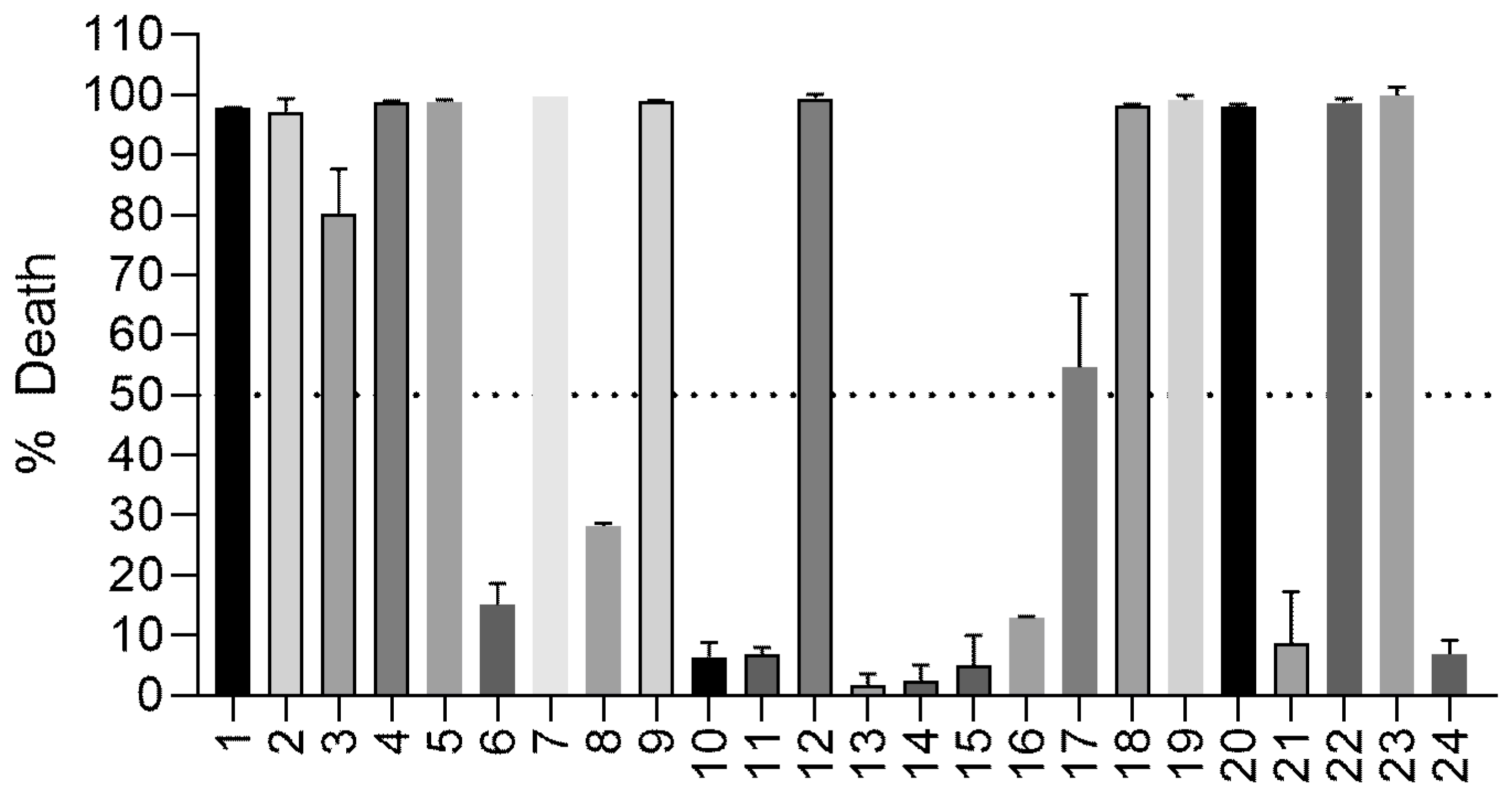
Activity of compounds **1**–**24** on *T. cruzi* amastigotes at 10 µg/mL. The dashed line indicates the 50% death threshold.

The trypanocidal activity of the compounds **1**–**24** was also evaluated against *T. cruzi* (intracellular forms) at 10 µg/mL (Figure 3).

The evaluation of compounds **1**–**24** against *T. cruzi* amastigotes revealed that several guaianolides and germacranolides, as well as eudesmanolide and eremophilenolide compounds, exhibited comparatively high activity at 10 µg/mL (Figure 3). Among them, eight guaianolides—estafiatin (**1**), grossheimin (**4**), cynaropicrin (**5**), anabasinyl-estafiatin (**9**), 8α-chloroacetoxygrossheimin (**12**), britanin (**18**), inuchinenolide C (**20**), and arglabin (**23**); four germacranolides—argolide (**2**), dihydroargolide (**3**), epoxyargolide (**7**), and stizolicin (**22**); and the eremophilenolide furanoeremophilan-14β,6α-olide (**17**) and the eudesmanolide pulchellin C (**19**) showed the highest levels of inhibition against *T. cruzi* at the concentration tested. In contrast, eight guaianolides—leucomisin (**6**), cytisinyl-estafiatin (**8**), austricin (**13**), 5β(H)-austricin (**14**), grossmisin (**15**), achillin (**16**), badkhyzin (**21**), and arborescin (**24**); one eudesmanolide—α-santonin (**10**); and the germacranolide 13α-cytisinylargolide (**11**) displayed comparatively low activity.

Overall, the antiparasitic activity of the studied STLs against *T. cruzi* generally followed trends like those observed for *T. b. brucei*. This suggests that certain structural features may influence activity across different *Trypanosoma* species. However, these observations are based on qualitative comparisons, and further studies are required to clarify the underlying mechanisms of action. Structural features affecting antiparasitic activity against one species are likely to influence activity against the other. Previous studies have suggested that STLs may interfere with thiol-dependent redox systems in trypanosomatids, including enzymes involved in trypanothione metabolism, such as thioredoxin–glutathione reductase and trypanothione S-transferase [14,17]. This hypothesis is further supported by computational target-identification studies that have proposed enzymes involved in parasite redox metabolism as potential molecular targets of trypanocidal STLs [18]. However, no direct biochemical assays were performed in the present study to confirm these specific molecular targets.

### 2.3. Cytotoxicity Determination

**Figure 4:**
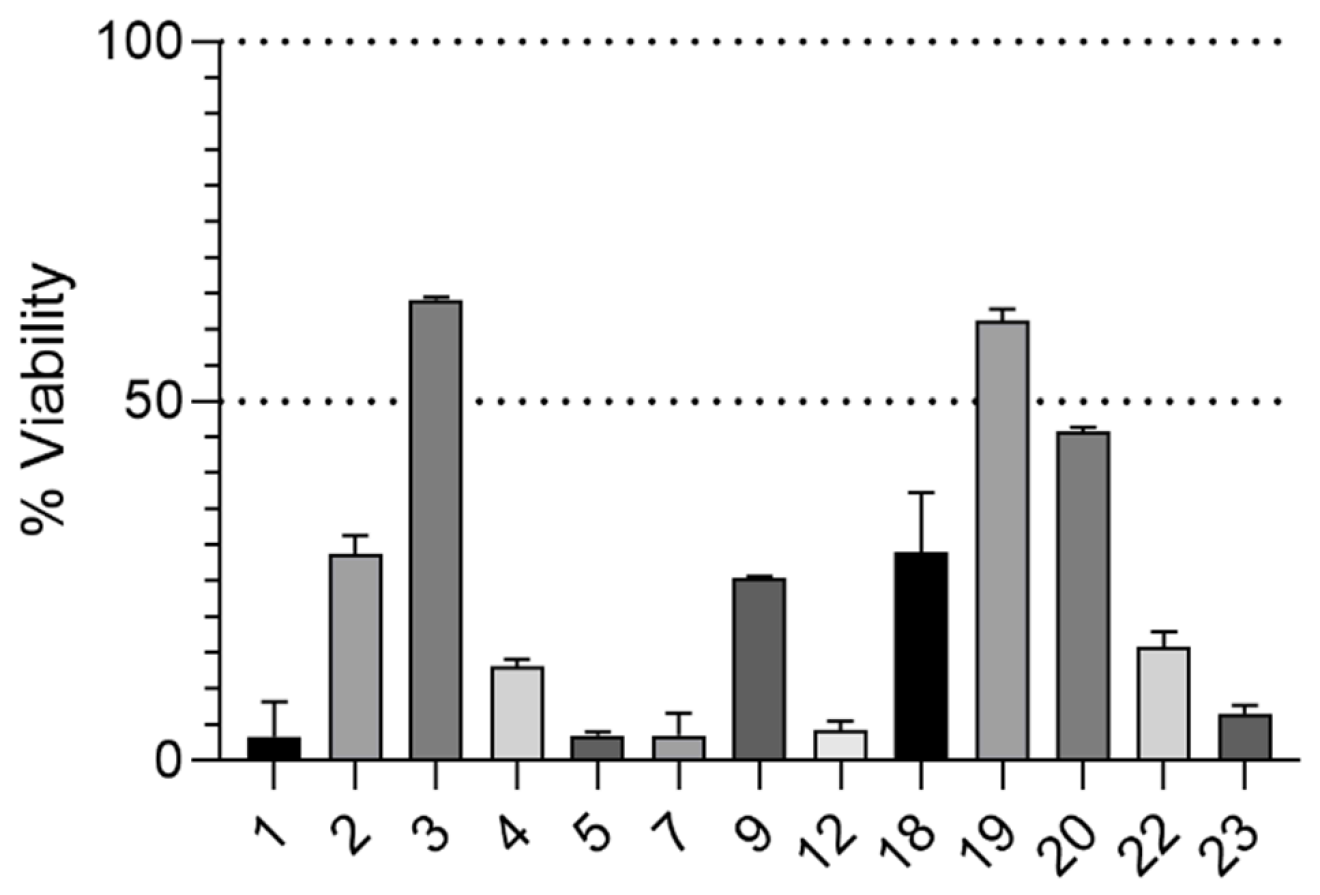
Cytotoxicity of estafiatin (**1**), argolide (**2**), dihydroargolide (**3**), grossheimin (**4**), cynaropicrin (**5**), epoxyargolide (**7**), anabasinyl-estafiatin (**9**), 8α-chloroacetoxygrossheimin (**12**), britanin (**18**), pulchellin C (**19**), inuchinenolide C (**20**), stizolicin (**22**), and arglabin (**23**) at a concentration of 10 µg/mL on Vero cells. The dashed line indicates the 50% viability threshold.

Based on the activity of sesquiterpene lactones **1**–**5**, **7**, **9**, **12**, **18**–**20**, and **22**–**23** against *T. cruzi* and *T. b. brucei* at 10 µg/mL, a cytotoxicity assessment was carried out at the same concentration (Figure 4).

Sesquiterpene lactones **3** and **19** showed viabilities higher than 50% in mammalian cells, while inhibiting 53.42% and 64.99% of *T. b. brucei* growth and 80.17% and 99.05% of *T. cruzi* growth, respectively, at 10 µg/mL. These findings may suggest that the presence of the exomethylene group (C11=C13) in the lactone ring could be associated with the biological activity and cytotoxicity of some STLs [19]. However, the available data would not allow the contribution of this structural feature to be clearly established, since its presence may not be essential for either activity or cytotoxicity. In this context, STLs containing both an exomethylene group and an epoxy moiety, such as estafiatin (**1**), could potentially exhibit both high biological activity and cytotoxicity. Interestingly, the anabasinyl derivative of estafiatin (**9**) showed a relatively higher percentage of Vero cell viability than the parent compound (**1**), which might suggest that amination at position C13 through a Michael-type addition could potentially reduce cytotoxicity. Nevertheless, further studies involving a broader range of structurally related compounds and quantitative structure–activity relationship analyses would be necessary to clarify whether these structural features could influence the biological activity and cytotoxicity of sesquiterpene lactones.

### 2.4. Determination of IC_50_ Values Against T. brucei brucei and T. cruzi

To further prioritize the most promising compounds identified during the initial screening, IC_50_ values were determined for selected STLs showing both measurable antiparasitic activity and comparatively lower cytotoxicity toward mammalian cells. Compounds **3** and **19** were selected based on their favorable preliminary selectivity profiles, representing the best balance between parasite inhibition and host-cell safety among the evaluated compounds. The aim of this analysis was to support early-stage candidate prioritization rather than to provide a comprehensive quantitative evaluation of all active compounds. Dose–response curves were therefore constructed for compounds **3** and **19** to determine their median inhibitory concentrations (IC_50_) against *T. b. brucei* (Figure S1) and *T. cruzi*.

The constructed dose–response curves confirm the dose-dependent inhibitory activity of dihydroargolide (**3**) and pulchellin C (**19**) against *T. b. brucei*. Although their potency is relatively lower than that of suramin, their markedly reduced cytotoxicity justified their selection for further evaluation within this early-stage screening study.

The IC_50_ values were determined using the same protocol as in the initial biological screening against *T. b. brucei* but employing a greater number of concentrations within a narrower range. The range was selected based on the inhibition percentages observed during the preliminary screening. Nonlinear regression analysis was performed using GraphPad Prism 5.01 software to calculate the IC_50_ values. Results are expressed as mean concentration ± standard deviation (SD) and summarized in Table 2.

According to the data in Table 2, pulchellin C (**19**) exhibits a relatively low IC_50_ of 2.51 ± 1.13 µg/mL (9.50 µM); however, this value is higher than that of the reference drug suramin (IC_50_ = 0.12 µM). These findings indicate that pulchellin C (**19**) exhibits relatively high activity against *T. b. brucei* under the tested conditions, and its high activity may be associated with the presence of the exomethylene group (C12=C13) in its lactone ring. Dihydroargolide (**3**) exhibited a comparatively higher IC_50_ value of 10.51 ± 1.72 µg/mL (41.98 µM). As expected from the primary screening results (Table 1), the antiparasitic activity of dihydroargolide (**3**) against *T. b. brucei* is lower than that of pulchellin C (**19**), which can be attributed to the hydrogenated C12-C13 bond. However, the activity displayed by dihydroargolide (**3**), together with its relatively low cytotoxicity, makes it an interesting candidate for further chemical modification. It is important to note that the selectivity indices observed in this study are generally below the threshold typically considered acceptable for advanced drug development (SI ≥ 10) [20]. This indicates that, despite detectable antiparasitic activity, the tested compounds require further structural optimization to improve their therapeutic window. Therefore, the present results should be interpreted as an early-stage screening outcome aimed at identifying preliminary hit compounds rather than fully optimized drug candidates.

A dose–response curve was also constructed to determine the median inhibitory concentrations (IC_50_) of dihydroargolide (**3**) and pulchellin C (**19**) on *T. cruzi* amastigotes (Figure S2). The IC_50_ values calculated for these compounds were 7.50 ± 0.23 µg/mL (29.96 µM) and 6.51 ± 0.14 µg/mL (24.63 µM), respectively. The CC_50_ values of compounds **3** and **19** on Vero cells were determined (Figure S3), and the selectivity index for each compound against *T. b. brucei* and *T. cruzi* was calculated (Table 3). It should be noted that IC_50_ values were determined only for selected compounds displaying both measurable antiparasitic activity and comparatively lower cytotoxicity. This approach was intended to prioritize the most promising candidates within an early-stage screening framework and does not constitute a comprehensive quantitative evaluation of all active compounds.

### 2.5. In Vitro Activity Against Leishmania amazonensis Promastigotes

The natural STLs and their derivatives were also tested for their activity on another protozoan: *L. amazonensis.* As shown in Table 4, estafiatin (**1**), grossheimin (**4**), cynaropicrin (**5**), epoxyargolide (**7**), anabasinyl-estafiatin (**9**), 8α-chloroacetoxygrossheimin (**12**) and arglabin (**23**) completely inhibited *L. amazonensis* promastigotes at a concentration of 10 µg/mL.

As shown in Table 4, eight guaianolides—estafiatin (**1**), grossheimin (**4**), cynaropicrin (**5**), anabasinyl-estafiatin (**9**), 8α-chloroacetoxygrossheimin (**12**), britanin (**18**), inuchinenolide C (**20**), and arglabin (**23**); four germacranolides—argolide (**2**), dihydroargolide (**3**), epoxyargolide (**7**), stizolicin (**22**); and the eudesmanolide pulchellin C (**19**) showed more than 95% inhibition of *L. amazonensis* at a concentration of 50 µg/mL.

Estafiatin (**1**) completely inhibited the growth of *L. amazonensis* at a concentration of 1 µg/mL, showing 7.4-fold-higher inhibition than its cytisinyl derivative (**8**) and 5.75-fold-higher inhibition than its anabasinyl derivative (**9**) at the same concentration. At 10 µg/mL, the difference in activity between the derivatives, compared to the natural estafiatin (**1**), changes: anabasinyl-estafiatin (**9**) also completely suppressed the growth of *L. amazonensis*, while cytisinyl-estafiatin (**8**) exhibited 7.51-fold-lower inhibition. As observed for *T. b. brucei*, this pattern suggests a structure-dependent biological response. At a concentration of 1 µg/mL, argolide (**2**) inhibited only 7.53% of *L. amazonensis* promastigotes, while dihydroargolide (**3**) showed 3.23-fold-higher antiparasitic activity, and 13α-cytisinylargolide (**11**) exhibited 2.73-fold-greater activity. This may indicate that the influence of the exomethylene group (C11=C13) in the lactone ring on antiparasitic activity against *L. amazonensis* is weaker than that observed against *T. b. brucei*. At the same time, epoxyargolide (**7**) did not inhibit *L. amazonensis* at 1 µg/mL, suggesting that, in contrast to its effect on *T. b. brucei*, the epoxy cycle at position C1-C10 would not influence the activity against *L. amazonensis*.

Grossheimin (**4**) showed no inhibitory activity against *L. amazonensis* at 1 µg/mL, whereas its 8α-chloroacetoxy derivative (**12**) inhibited more than 95% of the parasites at the same concentration. This finding, consistent with observations for *T. b. brucei*, indicates a strong correlation between acetylation of the hydroxyl group at C-8 and enhanced antiparasitic activity against *L. amazonensis*.

It is also noteworthy that arglabin (**23**), which showed 100% inhibition of *L. amazonensis* at 1 µg/mL, inhibited only 36.54% of *T. b. brucei* at the same concentration, further supporting the specificity of sesquiterpene lactone action against different species of parasites.

### 2.6. Determination of IC_50_ Values Against Leishmania amazonensis Promastigotes

The most active compounds were selected based on the results presented in Table 4. Compounds exhibiting more than 95% inhibition of parasite growth at a concentration of 1 µg/mL were selected for further evaluation. Accordingly, the guaianolides estafiatin (**1**), 8α-chloroacetoxygrossheimin (**12**), and arglabin (**23**) were identified as the most active compounds. For these compounds, dose–response curves were constructed to determine their IC_50_ against the promastigote form of *L. amazonensis* (Figure S4). The dose–response curves exhibit a sigmoidal profile, indicating a specific dose-dependent inhibition of parasite growth by estafiatin (**1**), 8α-chloroacetoxygrossheimin (**12**), arglabin (**23**), and amphotericin B. The curves for estafiatin (**1**), 8α-chloroacetoxygrossheimin (**12**), and arglabin (**23**) are shifted to the right relative to amphotericin B, indicating their lower potency compared to the widely used anti-leishmanial drug amphotericin B, whose curve is shifted furthest to the left.

Among the tested STLs, the curve for estafiatin (**1**) is closest to the comparison drug amphotericin B, demonstrating relatively high potency compared to the other γ-lactones. The curve for 8α-chloroacetoxygrossheimin (**12**) is slightly shifted to the right, while that of arglabin (**23**) appears furthest to the right, indicating its comparatively low potency. Additionally, the low cytotoxicity observed for these STLs makes them attractive candidates for further structural optimization.

It is also noteworthy that the presence of the exomethylene group (C11=C13) in the lactone ring of estafiatin (**1**), 8α-chloroacetoxygrossheimin (**12**), and arglabin (**23**) may contribute to their antiparasitic activity against *L. amazonensis*.

The dose–response curves visually corroborate the primary screening results (Table 4), highlighting the guaianolides estafiatin (**1**), 8α-chloroacetoxygrossheimin (**12**), and arglabin (**23**) as relatively active anti-leishmanial agents among the evaluated lactones.

According to the data in Table 5, estafiatin (**1**) exhibits a low IC_50_ (0.16 ± 0.13 µg/mL), but slightly higher than that of the reference drug amphotericin B (IC_50_ = 0.14 ± 0.09 µg/mL), highlighting estafiatin (**1**) as a potent inhibitor of *L. amazonensis*, likely due to the presence of an α-oriented epoxy cycle at position C3-C4 in its structure. Similarly, 8α-chloroacetoxygrossheimin (**12**) showed a low IC_50_ of 0.35 ± 0.18 µg/mL compared to the original grossheimin (**4**), whose IC_50_ ranges from 1 to 10 µg/mL. This observation suggests a strong correlation between acetylation of the hydroxyl group at C-8 and enhanced antiparasitic activity against *L. amazonensis*. Arglabin (**23**), in contrast, showed a relatively higher IC_50_ value of 0.58 ± 0.11 µg/mL, which may be attributed to the presence of a β-oriented epoxy cycle at position C1-C10 in its structure.

Overall, the calculated IC_50_ values confirm the high potential of the guaianolides estafiatin (**1**), grossheimin (**4**), 8α-chloroacetoxygrossheimin (**12**), and arglabin (**23**) against promastigotes. Notably, the activity of estafiatin (**1**) is comparable to that of the reference drug amphotericin B.

### 2.7. Cytotoxicity Determination of Active Compounds Against Leishmania amazonensis

The cytotoxicity of the guaianolides estafiatin (**1**), grossheimin (**4**), 8α-chloroacetoxygrossheimin (**12**), and arglabin (**23**) was determined on RAW macrophages by the 2,3-bis-(2-methoxy-4-nitro-5-sulfophenyl)-2H-tetrazolium-5-carboxanilide assay (XTT method). CC_50_ values were calculated for each compound and are presented in Table 6.

According to the data presented in Table 6, estafiatin (**1**) and 8α-chloroacetoxygrossheimin (**12**) exhibit relatively low CC_50_ values (<2 µg/mL), indicating comparatively high cytotoxicity toward the RAW macrophage cell line, which consists of host cells for the amastigote form of *Leishmania*. Consequently, this may limit the therapeutic potential of these compounds. In contrast, arglabin (**23**) shows a relatively higher CC_50_ value (2.50 ± 0.36 µg/mL), suggesting lower toxicity to non-target cells. Among the tested guaianolides, grossheimin (**4**) displayed the highest selectivity, with a CC_50_ of 4.04 ± 1.40 µg/mL.

### 2.8. In Vitro Activity Against Leishmania amazonensis Amastigotes

The activity of the most promising compounds was also tested on amastigotes of *L. amazonensis*. The results (IC_50_) and selectivity indexes are shown in Table 7:

Estafiatin (**1**) demonstrated a relatively low IC_50_ (0.68 ± 0.007 µg/mL) compared to the reference drug amphotericin B (IC_50_ < 0.924 µg/mL), characterizing estafiatin (**1**) as a potent inhibitor of intracellular *L. amazonensis* amastigotes. Its comparatively higher IC_50_ value relative to its activity against the promastigote form (IC_50_ = 0.16 ± 0.13 µg/mL) may be attributed to the need for the compound to penetrate the macrophage and then enter the parasitophorous vacuole to reach the amastigote. Grossheimin (**4**) showed an IC_50_ of 6.20 ± 2.46 µg/mL, suggesting low antiparasitic activity against *L. amazonensis* amastigotes. In contrast, arglabin (**23**) showed an IC_50_ value of 1.13 ± 0.006 µg/mL, which may be associated with the presence of a β-oriented epoxy cycle at C1-C10 in the structure.

According to the results obtained, estafiatin (**1**) (SI < 2.94) and arglabin (**23**) (SI = 2.22) were identified as the most selective compounds against amastigotes, in comparison to grossheimin (**4**) (SI < 1).

Elso et al. previously demonstrated that estafiatin showed in vitro activity against the bloodstream trypomastigote form of *T. cruzi*, with an IC_50_ value of 28.9 ± 4.1 µg/mL [21]. Comparable activity was observed against the intracellular amastigote stage, for which an IC_50_ value of 26.9 ± 4.3 µg/mL was determined. Mechanistic evaluation showed that estafiatin induced a pronounced inhibition of succinate dehydrogenase activity in *T. cruzi*, suggesting impairment of mitochondrial metabolism through disruption of the respiratory chain and the Krebs cycle. Furthermore, in vivo administration of estafiatin to *T. cruzi*-infected mice (1 mg/kg/day, intraperitoneally for five consecutive days) led to a significant reduction in parasitemia and attenuated inflammatory infiltrates and myocyte damage in muscle tissue [21].

In addition, four semisynthetic derivatives were obtained from estafiatin (**1**) (also known as estafietin), isolated from *Stevia alpina* (Asteraceae): 11βH,13-dihydroestafietin, 10(14)-epoxyestafietin, 11βH,13-methoxyestafietin, and 11βH,13-cianoestafietin. These compounds were evaluated for their antiprotozoal activity against *T. cruzi* and *L. braziliensis*. Among them, epoxyestafietin showed the highest potency against *T. cruzi* trypomastigotes and amastigotes, with IC_50_ values of 18.7 and 2.0 µg/mL, respectively. In contrast, estafiatin (**1**) and 11βH,13-dihydroestafietin displayed the greatest activity and selectivity against *L. braziliensis* promastigotes, with IC_50_ values of 1.0 and 1.3 µg/mL, respectively [21].

It is important to note that selectivity index (SI) values below 10 are often considered insufficient for further drug development. Accordingly, the in vitro results against *L. amazonensis* highlight that high antiparasitic activity against extracellular promastigotes does not necessarily translate into selective efficacy against intracellular amastigotes. Factors such as cellular uptake, stability within the acidic environment of the parasitophorous vacuole, and activity against metabolically distinct amastigote forms are likely to significantly influence the overall antiparasitic performance of these compounds [22,23,24].

The antiparasitic activity of STLs has been attributed to multiple mechanisms. A major structural determinant of their biological activity is the α-methylene-γ-lactone moiety, which undergoes Michael-type addition reactions with nucleophilic thiol groups present in proteins, enzymes, glutathione, and other cysteine-containing biomolecules. Modification of these targets may impair their biological function and disrupt cellular redox homeostasis [12]. Consistent with this, dehydroleucodine has been reported to interfere with the antioxidant defense system of *Trypanosoma cruzi*. The α-methylene-γ-lactone moiety is proposed to react with sulfhydryl groups of reducing molecules or enzymes, leading to reactive oxygen species accumulation and mitochondrial dysfunction [25]. More recently, Sardi et al. demonstrated that several STLs, including estafiatin (**1**) and arglabin (**23**), interfere with thiol-redox homeostasis in trypanosomatids using redox-reporter cell lines of *L. infantum, T. b. brucei* and HEK-293 cells. Estafiatin (**1**) selectively induced intracellular oxidation in both *L. infantum* and *T. brucei* without affecting mammalian HEK-293 cells, whereas arglabin (**23**) promoted oxidation in both parasites and mammalian cells, suggesting a less selective mechanism of action [26]. Together, these findings support the hypothesis that disruption of parasite thiol-redox metabolism may lead to mitochondrial dysfunction and ultimately contribute to the antiparasitic activity. In addition, STLs’ lactones have also been reported to modulate host immune responses. Their activity against *Leishmania* species, particularly *L. amazonensis*, has been associated with the suppression of interleukin-12 production [15]. This observation has prompted further interest in this class of compounds, as modulation of host immune responses may contribute, at least in part, to their antiparasitic effects.

It should be noted that the structure–activity relationships discussed in this study are based on qualitative comparisons within the tested compound set and do not represent a formal pharmacophore model. No molecular docking, computational modeling, or target-based biochemical assays were performed. Therefore, the proposed associations between specific structural features and antiparasitic activity should be considered as preliminary and hypothesis-generating, requiring further validation in future studies. No formal statistical significance testing was performed, as the present study was designed as an initial screening investigation. Additionally, certain assay validation parameters, such as Z-factor, were not determined in the present study, which may limit assessment of assay robustness.

## 3. Materials and Methods

The extracts from plants were obtained using CO_2_ extraction on the supercritical fluid extraction unit USFE-5/2 (manufactured by LLC “Goro-Engineering”, Rostov-on-Don, Russia, under license from the Austrian company “Natex”, Ternitz, Austria) with the following parameters (for all types of raw materials): pressure, 160 bar; temperature, 60 °C; and duration, 240 min [27]. Individual sesquiterpene lactones from a range of extracts were isolated using liquid column chromatography and flash chromatography on the Sepacore X50 Buchi unit (UCHI Labortechnik AG, Flawil, Switzerland).

The IR spectrum was recorded on an “Avatar 360 ESP” device by Thermo Nicolet (Madison, WI, USA) in KBr tablets.

HPLC was performed on an Agilent 1100 Series liquid chromatograph (Santa Clara, CA, USA) equipped with a QuatPump G1311A pump, a Rheodyne injection valve with a 20 µL loop, G1322A column holder, and G1314A UV detector. For all analyses, a Zorbax SB-C18 column (15 cm × 0.46 cm, 5 µm particles) (Agilent, Santa Clara, CA, USA) was used, and the solvents were methanol, acetonitrile, and water (analytical reagents with purity ≥ 99.9%, obtained from Sigma-Aldrich (Merck-Millipore, St. Louis, MO, USA) [28]. The maximum absorption in the UV spectrum at a wavelength of 205 nm was processed using ChemStation software (version A.10).

TLC was carried out using Silufol TLCP-AI A-UF plates from Imid (Krasnodar, Russia). The substances were analyzed by developing the plate with a 1% vanillin solution in H_2_SO_4_ and a saturated solution of KMnO_4_.

Column chromatography was performed on silica gel-grade KSKG (macroporous coarse-grained granular silica gel) (Qingdao Shuoyuan Silica Gel Technology Co., Ltd., Qingdao, China) with a ratio of substance to sorbent of 1:20. The purity of the isolated compounds was controlled using TLC and HPLC data. All experiments were performed in at least three independent replicates, and the results are presented as mean ± standard deviation (SD). Parasite strains used in this study were obtained from established laboratory collections and maintained under standard culture conditions, as described in previous studies. Detailed assay parameters, including multiplicity of infection and assay validation metrics (e.g., Z-factor), were not determined in the present study, which represents a limitation of experimental design.

### 3.1. Isolation of Sesquiterpene Lactones

Estafiatin (**1**) was isolated from the aboveground part of *Achillea nobilis* L. by a previously described method [29].

Argolide (**2**), dihydroargolide (**3**), and arglabin (**23**) were isolated from *Artemisia glabella* Kar. et Kir. by the method described by Adekenov et al. [27].

Grossheimin (**4**) and cynaropicrin (**5**) were isolated from the leaves and flower heads of *Chartolepis intermedia* Boiss. [26,30].

Leucomisin (**6**), austricin (**13**), 5β(H)-austricin (**14**), grossmisin (**15**), and achillin (**16**) were isolated from *Artemisia leucodes* Schrenk by the previously described method [31].

α-Santonin (**10**), found by us in more than 20 species of the *Artemisia* genus, was isolated from the aboveground parts of *Artemisia cina* Berg. by the previously described method [27].

Furanoeremophilan-14β,6α-olide (**17**) was isolated from the roots of *Ligularia macrophylla* (Ledeb.) DC. by the method previously described [32].

Britanin (**18**), pulchellin C (**19**), and inuchinenolide C (**20**) were isolated from the flower heads and leaves of *Inula caspica* Blume by the method published [33].

Badkhyzin (**21**) was isolated from the roots of *Ferula oopoda* Boiss. et Buhse by the method [34].

Stizolicin (**22**) was isolated by chloroform extraction from the hot aqueous extract of the aboveground part of *Stizolophus balsamita* (Lam.) Cass. ex Takht. [35].

Arborescin (**24**) was isolated from the aboveground part of *Artemisia austriaca* Jacq. by the method [36].

The compound library consisted of 24 sesquiterpene γ-lactones, including natural compounds isolated from plant sources and semisynthetic derivatives obtained through targeted chemical modifications such as amination, epoxidation, and acylation. The selection was based on the structural diversity within this class, the availability of well-characterized molecules, and the possibility to compare natural scaffolds with their corresponding derivatives in order to explore preliminary structure–activity relationships.

### 3.2. Synthesis of Epoxyargolide (***7***)

A total of 1.450 g of argolide (**2**) was dissolved in 50 mL of chloroform, and while stirring, 5 g of sodium carbonate was added, followed by a solution of acetic acid in chloroform. After 1.5 h, another 30 mL of the acetic acid solution was added, and the mixture was left for three days at room temperature. Thin-layer chromatography (TLC) showed, in addition to the starting compound (**2**), a spot with R_f_ 0.63 (ether–acetone). The reaction mixture was treated with an aqueous sodium carbonate solution, and the chloroform extract was dried over magnesium sulfate and evaporated under vacuum. Chromatographic separation of the residue (1.350 g) was carried out on a column with silica gel (grade L 40/100). A white crystalline substance with the molecular formula C_15_H_20_O_4_ (**7**) was obtained, with a melting point of 203–205 °C (ethyl acetate). [α]23D +40.3° (c, 1.19, CHCl_3_). Yield: 87%.

IR spectrum (KBr, ν_max_, cm^−1^): 2982, 2969, 2932 (C-H), 2863, 1761 (C=O γ-lactone), 1714 (C=O), 1662 (C=C), 1457, 1442, 1407, 1325, 1281, 1253, 1199, 1107, 1079, 1008 (-C-O-C-), 980, 949, 894, 818.

UV spectrum (EtOH, λ_max_, nm): 208.

Found, %: C 67.96; H 7.25; O 24.79. Calculated, %: C 68.16; H 7.63; O 24.21.

^1^H NMR (500 MHz, CDCl_3_, δ, m, J/Hz): 3.31 (1H, dd, J = 9.9, 5.6 Hz; H-1), 3.26 (1H, dd, J = 14.4, 5.6 Hz; H-2a), 2.23–2.18 (2H, m, H-2b, H-5a), 2.97–2.90 (1H, m, H-4), 1.45 (1H, qd, J = 13.72, 12.53, 11.7, 3.8 Hz, H-5b), 4.05 (1H, dd, J = 11.7, 3.0 Hz; H-6), 2.78 (1H, d, J = 10.6 Hz; H-7), 1.70–1.65 (1H, m, H-8a), 1.98–1.89 (1H, m, H-8b), 2.28 (1H, td, J = 3.65, 13.75 Hz, H-9a), 1.27–1.25 (1H, m, H-9b), 5.70 (1H, d, J = 1.4 Hz; H-13a), 6.28 (1H, d, J = 1.0 Hz; H-13b), 1.28 (3H, s, H-14), 1.16 (3H, d, J = 6.7 Hz; H-15).

^13^C NMR (125 MHz, CDCl_3_, δ, m, J/Hz): 59.0 (CH, C-1), 44.0 (CH_2_, C-2), 210.2 (C, C-3), 40.0 (CH_2_, CH, C-4), 39.9 (CH_2_, C-5), 80.9 (CH, C-6), 44.1 (CH, C-7), 33.8 (CH_2_, C-8), 39.5 (CH_2_, C-9), 59.1 (C, C-10), 140.2 (C, C-11), 169.3 (C, C-12), 123.8 (CH_2_, C-13), 18.2 (CH_3_, C-14), 15.7 (CH_3_, C-15).

### 3.3. Synthesis of Cytisinyl-estafiatin (***8***)

To 0.25 g (0.001 mol) of estafiatin (**1**) dissolved in 3 mL of methanol, 0.228 g (0.0012 mol) of cytisine was added. The reaction mixture was stirred for 18 h at room temperature. The resulting oil was dissolved in ethyl acetate, and upon adding petroleum ether, a precipitate formed, which was recrystallized from EtOH, yielding a white crystalline substance with the composition C_26_H_32_O_4_N_2_ (**8**) and a melting point of 214–217 °C. Yield: 250 mg (57%).

IR spectrum (KBr, ν_max_, cm^−1^): 2973, 2967, 2945, 2934, 2799 (C-H), 1757 (carbonyl γ-lactone), 1652 (C=N), 1591, 1577, 1568 (C=C), 1462, 1450, 1378, 1329, 1300, 1209 (epoxy cycle), 1191 (C-N), 1075, 1048 (CH2), 985, 957, 826, 802 (C-O-C).

UV spectrum (EtOH, λ_max_, nm): 203.

Found, %: C 71.58; H 7.42; N 6.36. Calculated, %: C 71.56; H 7.34; N 6.42.

^1^H NMR (500 MHz, CDCl_3_, δ, m, J/Hz): 1.93 (1H, m, H-1), 1.79 (1H, m, H-2a), 1.96 (1H, m, H-2b), 3.29 (1H, s, H-3), 1.86 (1H, m, H-5), 3.81 (1H, t, J = 10.0, H-6), 1.93 (1H, m, H-7), 2.20 (1H, dd, J = 20.0, 9.7, 2.5, H-8a), 2.79 (1H, dd, J = 9.7, 5.7, H-8b), 1.68 (1H, ddd, J = 13.96, 10.52, 6.0, H-9a), 2.00 (1H, dd, J = 13.96, 7.3, H-9b), 2.10 (1H, dd, J = 11.0, 8.5, H-11), 2.47 (1H, dd, J = 13.5, 10.0, H-13a), 2.72 (1H, dd, J = 13.5, 3.5, H-13b), 4.74 (1H, s, H-14a), 4.79 (1H, s, H-14b), 1.50 (3H, s, H-15), 6.44 (1H, dd, J = 9.0, 1.4, H-3′), 7.25 (1H, dd, J = 9.0, 6.8, H-4′), 5.96 (1H, dd, J = 6.8, 1.1, H-5′), 2.95 (1H, s, H-7′), 1.74 (1H, m, H-8′a), 1.88 (1H, m, H-8′b), 2.42 (1H, s, H-9′), 3.87 (1H, dd, J = 15.0, 10.0, H-10′a), 4.02 (1H, d, J = 15.0, H-10′b), 2.49 (1H, dd, J = 11.0, H-11′a), 2.86 (1H, d, J = 11.0, H-11′b), 2.25 (1H, dd, J = 11.0, 2.0, H-13′a), 2.88 (1H, dd, J = 11.0, H-13′b).

^13^C NMR (125 MHz, CDCl_3_, δ, m, J/Hz): 30.6 (d, C-1), 31.1 (t, C-2), 63.3 (d, C-3), 66.0 (s, C-4), 48.0 (d, C-5), 80.8 (d, C-6), 44.4 (d, C-7), 44.5 (t, C-8), 32.8 (t, C-9), 146.8 (s, C-10), 50.4 (d, C-11), 177.0 (s, C-12), 58.6 (t, C-13), 114.3 (q, C-14), 18.8 (q, C-15), 163.3 (s, C-2′), 116.9 (d, C-3′), 138.5 (d, C-4′), 104.6 (d, C-5′), 151.3 (s, C-6′), 35.2 (d, C-7′), 25.8 (t, C-8′), 28.2 (d, C-9′), 50.0 (t, C-10′), 62.5 (t, C-11′), 59.5 (t, C-13′).

### 3.4. Synthesis of Anabasinyl-estafiatin (***9***)

To 0.25 g (0.001 mol) of estafiatin (**1**) dissolved in 3 mL of methanol, 0.2 g (0.0012 mol) of anabasine was added. The reaction mixture was stirred for 26 h at room temperature. The resulting oil was dissolved in ethyl acetate, and upon adding petroleum ether, a precipitate formed, which was recrystallized from EtOH, yielding a white crystalline substance with the composition C_25_H_32_O_3_N_2_ (**9**). Yield: 330 mg (88%).

IR spectrum (KBr, ν_max_, cm^−1^): 2984, 2947, 2932, 2912 (C-H), 1774 (C=O γ-lactone), 1633 (C=N), 1591, 1577, 1568 (C=C), 1453, 1443, 1427, 1327, 1303, 1209 (epoxy cycle), 1199 (C-N), 1054, 1024, 1003 (CH2), 985, 961, 826, 802 (C-O-C).

UV spectrum (EtOH, λ_max_, nm): 203.

Found, %: C 73.51; H 7.86; N 6.85. Calculated, %: C 73.53; H 7.84; N 6.86.

^1^H NMR (500 MHz, CDCl_3_, δ, m, J/Hz): 3.10 (1H, m, H-1), 2.18 (2H, m, H-2a, H-2b), 3.34 (1H, s, H-3), 2.30 (1H, m, H-5), 3.95 (1H, t, J = 10.5, H-6), 2.35 (1H, m, H-7), 2.12 (2H, m, H-8a, H-8b), 2.22 (2H, m, H-9a, H-9b), 1.55 (1H, m, H-11), 2.31 (1H, dd, J = 13.53, 2.72, H-13a), 2.66 (1H, dd, J = 13.53, 6.87, H-13b), 4.87 (1H, s, H-14a), 4.92 (1H, s, H-14b), 1.53 (3H, s, H-15), 1.69 (1H, m, H-2a′, H-3a′), 1.72 (1H, m, H-2b′, H-3b′), 1.78 (1H, d, J = 1.8, H-4a′), 2.30 (1H, d, J = 1.8, H-4b′), 1.33 (1H, m, H-5a′, H-5b′), 2.84 (1H, d, J = 10.5, H-6a′), 2.93 (1H, d, J = 10.0, H-6b′), 8.5 (1H, d, J = 1.8, H-8′), 7.66 (1H, d, J = 7.8, H-10′), 7.25 (1H, dd, J = 7.8, 1.8, H-11′), 8.48 (1H, dd, J = 4.8, 1.8, H-12′).

^13^C NMR (125 MHz, CDCl_3_, δ, m, J/Hz): 30.2 (d, C-1), 30.7 (t, C-2), 63.5 (d, C-3), 66.3 (s, C-4), 44.7 (d, C-5), 80.8 (d, C-6), 42.6 (d, C-7), 44.7 (t, C-8), 32.9 (t, C-9), 147.1 (s, C-10), 51.0 (d, C-11), 178.0 (s, C-12), 54.0 (t, C-13), 114.4 (q, C-14), 18.7 (q, C-15), 66.1 (d, C-2′), 36.5 (t, C-3′), 24.6 (t, C-4′), 25.7 (t, C-5′), 51.9 (t, C-6′), 148.8 (d, C-8′), 140.3 (s, C-9′), 135.6 (d, C-10′), 123.6 (d, C-11′), 149.6 (d, C-12′).

### 3.5. Synthesis of 13α-Cytisinylargolide (***11***)

To 1 g (0.0040 mol) of argolide (**2**) dissolved in 15 mL of methyl alcohol, 0.912 g (0.0048 mol) of cytisine alkaloid was added. The reaction mixture was stirred at room temperature for 24 h. After the reaction was completed, the alcohol was removed using a rotary evaporator. The reaction mixture was extracted with chloroform (1:8). The chloroform layer containing the target substance was separated, dried over magnesium sulfate, evaporated under vacuum, and the residue was chromatographed on a column with silica gel. Elution of the column with the reaction mixture using a petroleum ether and ethyl acetate (1:9) system resulted in the isolation of a colorless crystalline substance with the molecular formula C_26_H_34_O_4_N_2_ (**11**) and a melting point of 185–187 °C. Yield: 45%. R_f_ = 0.63 (ethyl acetate-ethyl alcohol = 2:1).

IR spectrum (KBr, ν_max_, cm^−1^): 3030, 2994, 2925, 2884, 2852, 2796, 2360, 2341, 1766 (C=O γ-lactone), 1705, 1658, 1581, 1569, 1547, 1472, 1449, 1424, 1373, 1360, 1336, 1311, 1238, 1215, 1202, 1175, 1065, 980, 802, 711, 675, 558.

UV spectrum (EtOH, λ_max_, nm): 203, 234, 311.

Found, %: C 71.02; H 7.74; N 6.37. Calculated, %: C 71.23; H 7.76; N 6.39.

^1^H NMR (500 MHz, CDCl_3_, δ, m, J/Hz): 5.29 (1H, t, J = 8.5, H-1), 2.98 (3H, m, H-7′, H-2a, H-2b), 2.66 (1H, m, H-4), 1.40 (1H, ddd, J = 14.2, 9.9, 3.3, H-5a), 1.94 (4H, m, H-9b, H-8′b, H-7, H-5b), 3.59 (1H, d, J = 9.0, H-6), 1.56 (1H, td, J = 14.2, 13.0, 3.9, H-8a), 1.09 (1H, dt, J = 14.3, 3.4, H-8b), 1.75 (2H, m, H-8′a, H-9a), 2.25 (1H, ddd, J = 11.6, 5.2, 4.0, H-11), 2.44 (1H, dd, J = 12.7, 11.7, H-13a), 2.69 (1H, dd, J = 12.9, 5.2, H-13b), 1.48 (3H, s, H-15), 1.03 (3H, d, J = 6.7, H-15), 6.46 (1H, dd, J = 9.0, 1.4, H-3′), 7.3 (1H, dd, J = 9.0, 6.9, H-4′), 6.0 (1H, dd, J = 6.9, 1.3, H-5′), 2.51 (1H, m, H-9′), 3.94 (1H, dd, J = 15.3, 6.6, H-10′a), 4.02 (1H, d, J = 15.5, H-10′b), 2.57 (1H, d, J = 11.0, H-11′a), 2.82 (1H, m, H-11′b), 2.29 (1H, d, J = 10.8, H-13′a), 2.89 (1H, m, H-13′b).

^13^C NMR (125 MHz, CDCl_3_, δ, m, J/Hz): 118.0 (CH, C-1), 42.2 (CH_2_, C-2), 208.9 (C=O, C-3), 39.9 (CH, C-4), 39.0 (CH2, C-5), 84.3 (CH, C-6), 40.4 (CH, C-7), 33.0 (CH_2_, C-8), 39.6 (CH_2_, C-9), 140.5 (C, C-10), 47.1 (CH, C-11), 177.8 (C=O, C-12), 58.9 (CH_2_, C-13), 15.9 (CH_3_, C-14), 18.3 (CH_3_, C-15), 163.4 (C=O, C-2′), 117.1 (CH, C-3′), 139.0 (CH, C-4′), 104.4 (CH, C-5′), 151.5 (C, C-6′), 35.1 (CH, C-7′), 25.7 (CH_2_, C-8′), 28.2 (CH, C-9′), 50.3 (CH_2_, C-10′), 62.6 (CH_2_, C-11′), 58.7 (CH_2_, C-13′).

### 3.6. Synthesis of 8α-Chloroacetoxygrossheimin (***12***)

To a solution of grossheimin (**4**) (500 mg, 1.9 mmol) in pyridine (5 mL), chloroacetic anhydride (0.65 g, 3.8 mmol, 2 equivalents) was added. The reaction was carried out by stirring at room temperature for 20 min. The reaction mixture was then transferred to a separatory funnel, and chloroform (15 mL) and a 5% aqueous hydrochloric acid solution were added. The treatment was repeated 3 times (until a neutral medium was achieved). Then, the organic layer was dried over MgSO_4_, filtered after 1 h, and the filtrate was evaporated under a vacuum. The obtained residue was recrystallized from ethanol, resulting in the isolation of 490 mg (87% yield) of a crystalline substance with a brownish hue, composition of C_17_H_19_O_5_Cl (**12**), and melting point of 162–164 °C (in ethanol). R_f_ = 0.41 (ethyl acetate-ethyl alcohol = 4:1).

IR spectrum (cm^−1^): 1754 (C=O γ-lactone), 1723 (C=O), 1647 (C=C), 1453, 1417, 1360, 1338, 1316, 1296, 1278, 1240 (C=O ester group), 1207, 1143, 821 (C–Cl).

UV spectrum (EtOH, λ_max_, nm): 205 (Ɛ 1.225), 258 (Ɛ 0.5585).

Found, %: C 59.39; H 5.41; Cl 12.15. Calculated, %: C 60.26; H 5.6; Cl 10.5.

^1^H NMR (500.16 MHz, CDCl_3_), δ (m, J/Hz): 6.36 (d, J = 3.3 Hz, 1H), 5.92 (d, J = 2.9 Hz, 1H), 5.15 (s, 1H), 5.01 (m, 1H), 4.88 (s, 1H), 4.14 (d, J = 0.8 Hz, 1H), 4.06 (dd, J = 9.1 Hz, 1H), 3.38 (m, 1H), 3.16 (m, 1H), 3.00 (dd, J = 13.0, 5.8 Hz, 1H), 2.56 (dd, J = 19.1, 8.7 Hz, 1H), 2.49 (dd, J = 19.2, 3.4 Hz, 1H), 2.37–2.24 (m, 3H), 1.25 (d, J = 6.7 Hz, 3H).

^13^C NMR (127.76 MHz, CDCl_3_), δ (m, J/Hz): 218.6, 169.37, 166.3, 141.7, 135.3, 125.5, 117.4, 82.1, 76.3, 51.0, 46.2, 43.2, 43.1, 40.1, 40.1, 14.9, 7.0.

Chemical structures of the compounds are shown in Figure 1.

### 3.7. Cytotoxicity on Vero Cells

The cytotoxicity of the compounds was determined using Vero cell cultures (Vero ATCC CCL-81, Manassas, VA, USA) in 96-well plates. EMEM medium was used for cell cultivation, supplemented with 200 μM L-glutamine, 10% fetal bovine serum, 100 U/mL penicillin, 100 μg/mL streptomycin, and 25 μg/mL amphotericin B. The cells were incubated at 37 °C in a 5% CO_2_ atmosphere. The substances were added 24 h after starting the cell culture. All samples were previously dissolved in dimethyl sulfoxide (DMSO).

The samples were diluted with the nutrient medium and then filtered through membrane filters with a pore diameter of 0.22 μm. The concentration of the samples was 3.0 and 5.0 μg/mL. Control groups included cells containing similar concentrations of DMSO. The cell cultures were incubated with the samples at 37 °C for 72 h, and then 20 μL of prepared 3-(4,5-dimethylthiazol-2-yl)-2,5-diphenyl-2H-tetrazolium bromide (MTT) solution (initial concentration 5 mg/mL in phosphate buffer) was added to the medium.

The MTT incubation was carried out for 4 h at 37 °C. Removing the medium led to the formation of insoluble formazan, which was extracted by adding 100 μL of dimethyl sulfoxide and incubating in the dark at room temperature for 15 min. Optical density was measured using the StatFax 2100 spectrophotometer at 492 nm.

### 3.8. Activity Against the Blood Form of Trypanosoma brucei brucei

The initial concentration of parasites was adjusted to 3.5 × 10^4^ parasites/mL, and 200 μL was added to sterile 96-well plates. Then, the diluted compounds were added, resulting in final concentrations of 0.1 μg/mL, 1 μg/mL, and 10 μg/mL in duplicate wells. The plate was incubated for 72 h at 37 °C, with 5% CO_2_. After the incubation period, parasites were counted using a Beckman Z2 Coulter counter. The concentrations of 0.1, 1, and 10 μg/mL were selected as part of a primary screening strategy commonly used for early antiparasitic evaluation, allowing for rapid identification of highly active compounds across a broad activity range. Compounds showing >95% inhibition at 10 μg/mL were considered active and prioritized for further IC_50_ determination using a more detailed concentration–response analysis.

### 3.9. Activity Against Amastigotes of Trypanosoma cruzi

Vero cells (5 × 10^3^/well) were infected with *T. cruzi* Tulahuen strain trypomastigotes expressing β-galactosidase (MOI: 10). After overnight incubation at 37 °C and 5% CO_2_, non-infecting parasites were washed out, and infected cells were incubated with sesquiterpene lactones 1–24 at a concentration of 10 µg/mL. After 5 days, the cells were lysed with 1% Nonidet P-40, and chlorophenol red-β-D-galactopyranoside (CPRG) (100 µM) was added as a substrate for β-galactosidase. The plate was incubated for 4 h at 37 °C, and β-galactosidase activity was determined by measuring absorbance at 570 nm. The percentage of inhibition for each concentration of the compound was calculated as follows: 100 − {[(absorbance of treated infected cells)/(absorbance of untreated infected cells)] × 100}.

### 3.10. Activity Against Promastigotes of Leishmania amazonensis

The parasite concentration was adjusted to 5 × 10^5^ promastigotes/well in the stationary phase and then diluted in RPMI medium without phenol red. The diluted parasites were distributed into 96-well plates. The compounds were dissolved in dimethyl sulfoxide (DMSO) and diluted in RPMI medium without phenol red, achieving final concentrations of 50, 10, and 1 μg/mL in duplicate wells. The final concentration of DMSO in the medium, used for dissolving the compounds, did not affect the parasite viability during the analysis. High concentrations of DMSO were used as a negative control in the medium. After 48 h at 37 °C, 50 μL of the XTT reagent was added to the parasite culture with and without the compounds, and the plates were incubated at 37 °C for 4 h. The viability of the promastigotes was determined in a microplate reader (Tecan, Männedorf, Switzerland) at a wavelength of 450 nm.

### 3.11. Cytotoxicity on Macrophages

To assess the cytotoxic effect of the compounds on the RAW macrophage cell line (RAW ATCC -264.7), a colorimetric assay was used. This method evaluates cell viability based on the ability of viable cells to reduce the tetrazolium salt 2,3-bis-(2-methoxy-4-nitro-5-sulfophenyl)-5-(phenylamino)-carbonyl-2H-tetrazolium hydroxide (XTT), a reaction dependent on mitochondrial enzymatic activity. The initial cell concentration was adjusted to 4 × 10^4^ cells/well and added to sterile 96-well plates. Serial dilutions of the guaianolides estafiatin (**1**), grossheimin (**4**), 8α-chloroacetoxygrossheimin (**12**), and arglabin (**23**) were added in duplicate. Plates were incubated at 37 °C in 5% CO_2_ for 48 h. After incubation, the culture medium was carefully removed and replaced with PBS (pH 7.2). Then, 50 µL of the XTT reagent was added to each well, and plates were incubated under the same conditions for 4 h. Cell viability was determined using a microplate reader (Tecan) at 450 nm. CC_50_ values were calculated using GraphPad Prism version 5.01. Results are presented in Table 7.

### 3.12. Activity Against Leishmania amazonensis Amastigotes

To determine the activity of compounds against intracellular amastigotes of *Leishmania amazonensis*, macrophages (RAW cells) were counted using a Neubauer chamber. Cell viability was determined using Trypan blue, and the concentration of cells was adjusted to 1.15 × 10^5^ cells/mL. Then, 100 µL of the cell suspension was added to 16-well plates (LAB Tek, Waltham, MA, USA), and the plate was incubated at 37 °C in 5% CO_2_ for 4 h to allow the cells to adhere to the bottom of the well. After 4 h, the infection rate was set at 20 parasites per cell using *L. amazonensis* promastigotes in the stationary phase. The plate was incubated at 34 °C in 5% CO_2_ for 24 h. The following day, the wells were thoroughly washed to remove free parasites, and the plate was incubated for an additional 24 h under the same conditions. After this incubation period, serial dilutions of the compounds were added, and the plate was incubated for another 48 h. Finally, the culture medium was removed from the plate, and the chamber was removed to expose the fixed amastigotes at the bottom of the wells. The fixed amastigotes were stained with Tincion 15 (Biopur, Bad Bubendorf, Switzerland) and then counted using an optical microscope at 100× magnification with immersion oil. The percentage reduction of infection (% RI) was calculated using the following equation and the IC_50_:

$$\%\;\mathrm{RI}\;=\;\frac{IS\;compound}{IS\;control}\times 100\;\mathrm{IS}\;=\;\%\;\mathrm{infected}\;\mathrm{macrophages}\;\times \;\frac{\#\;amastigotes}{\#\;macrophages}$$

## 4. Conclusions

As a result of the evaluation of natural sesquiterpene lactones isolated from Kazakh plant species belonging to the Apiaceae and Asteraceae families and semisynthetic derivatives, two compounds, dihydroargolide (**3**) and pulchellin C (**19**), exhibited high activity against *T. b. brucei* trypomastigotes and *T. cruzi* amastigotes. Among the evaluated compounds, pulchellin C (**19**) showed the highest selectivity against both trypanosomes, with an IC_50_ of 2.51 ± 1.13 µg/mL (9.50 µM) against *T. b. brucei* and a selectivity index of 3.96, as well as an IC_50_ of 6.51 ± 0.14 µg/mL (24.63 µM) against *T. cruzi* (SI=1.53). Moreover, estafiatin (**1**), 8α-chloroacetoxygrossheimin (**12**), and arglabin (**23**) displayed the strongest activity against *L. amazonensis* promastigotes, with IC_50_ values of 0.16 ± 0.13, 0.35 ± 0.18, and 0.58 ± 0.11 µg/mL, respectively. In addition, estafiatin (**1**) demonstrated the strongest activity against *L. amazonensis* amastigotes, with an IC_50_ of 0.68 ± 0.007 µg/mL and moderate selectivity (SI < 2.94), while arglabin (**23**) also showed promising activity with an IC_50_ of 1.13 ± 0.006 µg/mL and an SI = 2.22. Additionally, structure–activity relationship analysis revealed the following: Michael addition of estafiatin (**1**) to the exomethylene group C11=C13 leads to a decrease in the antiparasitic activity of its derivatives, cytisinylestafiatin (**8**) and anabasinylestafiatin (**9**), against *T. b. brucei* and *L. amazonensis* promastigotes, as well as a decrease in cytotoxicity (compound **9**).

Hydrogenation of argolide (**2**) at the exomethylene group C11=C13 decreases both cytotoxicity and antiparasitic activity of dihydroargolide (**3**) against *T. b. brucei,* but it increases its antiparasitic activity against *L. amazonensis* promastigotes.

Epoxidation of argolide (**2**) at position C1-C10 leads to an increase in cytotoxicity and antiparasitic activity of epoxyargolide (**7**) against *T. b. brucei*.

Michael addition of argolide (**2**) to the exomethylene group C11=C13 leads to a decrease in antiparasitic activity of 13α-cytisinylargolide (**11**) against *T. b. brucei* and against *L. amazonensis* promastigotes.

Acylation of the hydroxyl group of grossheimin (**4**) at C-8 leads to an increase in the antiparasitic activity of 8α-chloroacetoxygrossheimin (**12**) against *T. b. brucei* and *L. amazonensis* promastigotes, as well as an increase in its cytotoxicity.

Further studies will focus on determining the possible mechanisms of action of the most active sesquiterpene lactones. This information will guide the targeted chemical modifications of estafiatin (**1**), leucomisin (**6**), 8α-chloroacetoxygrossheimin (**12**), pulchellin C (**19**), and arglabin (**23**) to improve their antiparasitic activity and selectivity using molecular docking methods.

## Figures and Tables

**Table 1:** Bio-screening of sesquiterpene lactones and their derivatives on bloodstream forms of *T. b. brucei*.

Compound	Inhibition (%)		
	10 µg/mL ± SD	1 µg/mL ± SD	0.1 µg/mL ± SD
Estafiatin (**1**)	99.24 ± 0.18	72.91 ± 1.83	44.33 ± 1.83
Argolide (**2**)	98.94 ± 0.29	10.82 ± 6.98	NI
Dihydroargolide (**3**)	53.42 ± 0.36	13.16 ± 5.51	NI
Grossheimin (**4**)	98.75 ± 0.41	4.07 ± 3.67	NI
Cynaropicrin (**5**)	99.29 ± 0.24	4.07 ± 0.01	1.47 ± 0.03
Leucomisin (**6**)	4.71 ± 6.20	2.77 ± 1.83	2.77 ± 0.02
Epoxyargolide (**7**)	97.43 ± 1.84	99.18 ± 0.03	NI
Cytisinyl-estafiatin (**8**)	4.77 ± 3.67	NI	NI
Anabasinyl-estafiatin (**9**)	99.31 ± 0.52	5.36 ± 1.83	NI
α-santonin (**10**)	4.07 ± 0.36	NI	NI
13α-Cytisinylargolide (**11**)	6.66 ± 3.67	9.26 ± 0.18	NI
8α-Chloroacetoxygrossheimin (**12**)	99.18 ± 0.63	45.63 ± 1.28	4.07 ± 3.67
Austricin (**13**)	35.24 ± 3.67	31.34 ± 12.85	39.13 ± 1.83
5β(H)-austricin (**14**)	2.77 ± 1.83	NI	NI
Grossmisin (**15**)	1.47 ± 0.55	NI	NI
Achillin (**16**)	27.45 ± 11.02	1.47 ± 0.01	NI
Furanoeremophilan-14β,6α-olide (**17**)	10.56 ± 5.51	NI	NI
Britanin (**18**)	98.77 ± 0.37	19.65 ± 14.69	9.26 ± 3.67
Pulchellin C (**19**)	64.99 ± 14.69	28.74 ± 5.51	18.35 ± 1.83
Inuchinenolide C (**20**)	95.33 ± 2.54	36.54 ± 1.83	19.65 ± 11.02
Badkhyzin (**21**)	36.54 ± 12.85	31.34 ± 5.51	33.94 ± 1.83
Stizolicin (**22**)	89.42 ± 3.22	11.86 ± 0.18	9.26 ± 7.34
Arglabin (**23**)	99.16 ± 0.12	36.54 ± 12.85	33.94 ± 9.18
Arborescin (**24**)	5.36 ± 1.83	NI	NI

NI: Inhibition not observed.

**Table 2:** In vitro activity of dihydroargolide (**3**) and pulchellin C (**19**) against the bloodstream form of *Trypanosoma brucei brucei*.

Compounds	IC_50_ (µg/mL) ± SD (µM)
Dihydroargolide (**3**)	10.51 ± 1.72 (41.98)
Pulchellin C (**19**)	2.51 ± 1.13 (9.50)
Suramin	0.12 ± 0.02 (*)

(*) The IC_50_ value of suramin is expressed in µM.

**Table 3:** CC_50_ values and selectivity indexes on *T. brucei brucei* and *T. cruzi*.

Compound	CC_50_ (µg/mL) ± SD	SI *T. b. brucei*	SI *T. cruzi*
Dihydroargolide (**3**)	12.43 ± 0.90	1.18	1.66
Pulchellin C (**19**)	9.95 ± 0.05	3.96	1.53

**Table 4:** Bio-screening of sesquiterpene lactones and their derivatives against *Leishmania amazonensis* promastigotes.

Compound	Inhibition (%)		
	50 µg/mL ± SD	10 µg/mL ± SD	1 µg/mL ± SD
Estafiatin (**1**)	98.68 ± 0.28	100 ± 0.05	100 ± 0.91
Argolide (**2**)	100 ± 0.77	79.36 ± 1.09	7.53 ± 13.09
Dihydroargolide (**3**)	100 ± 0.21	81.57 ± 3.79	24.32 ± 5.97
Grossheimin (**4**)	100 ± 2.30	100 ± 5.01	NI
Cynaropicrin (**5**)	100 ± 0.96	100 ± 1.34	24.73 ± 7.59
Leucomisin (**6**)	17.87 ± 4.54	11.80 ± 4.28	8.09 ± 7.13
Epoxyargolide (**7**)	100 ± 2.47	100 ± 0.91	NI
Cytisinyl-estafiatin (**8**)	22.80 ± 9.07	13.31 ± 0.01	13.52 ± 4.45
Anabasinyl-estafiatin (**9**)	100 ± 0.63	100 ± 1.20	17.39 ± 7.34
α-santonin (**10**)	37.81 ± 2.23	10.10 ± 5.01	12.81 ± 3.94
13α-Cytisinylargolide (**11**)	37.70 ± 5.50	12.67 ± 1.40	20.60 ± 1.14
8α-Chloroacetoxygrossheimin (**12**)	100 ± 0.31	100 ± 0.35	95.50 ± 0.80
Austricin (**13**)	29.29 ± 4.37	21.13 ± 3.68	14.71 ± 0.73
5β(H)-austricin (**14**)	7.91 ± 1.17	16.69 ± 13.01	NI
Grossmisin (**15**)	40.49 ± 8.33	34.92 ± 0.03	31.84 ± 0.14
Achillin (**16**)	35.14 ± 7.90	30.51 ± 14.10	25.95 ± 4.76
Furanoeremophilan-14β,6α-olide (**17**)	34.94 ± 1.12	29.87 ± 1.29	16.77 ± 4.14
Britanin (**18**)	100 ± 5.13	79.11 ± 2.62	25.39 ± 5.74
Pulchellin C (**19**)	100 ± 0.60	59.05 ± 1.24	16.77 ± 4.14
Inuchinenolide C (**20**)	100 ± 1.44	62.46 ± 1.05	32.45 ± 2.52
Badkhyzin (**21**)	38.45 ± 6.88	42.79 ± 0.51	14.97 ± 0.15
Stizolicin (**22**)	98.46 ± 1.57	32.81 ± 5.90	9.59 ± 3.56
Arglabin (**23**)	100 ± 3.23	100 ± 2.34	100 ± 1.00
Arborescin (**24**)	33.01 ± 2.10	20.81 ± 3.40	NI

NI: Inhibition not observed.

**Table 5:** In vitro activity of estafiatin (**1**), 8α-chloroacetoxygrossheimin (**12**), and arglabin (**23**) against *Leishmania amazonensis* promastigotes.

Compounds	IC_50_ (µg/mL) ± SD
Estafiatin (**1**)	0.16 ± 0.13
8α- chloroacetoxygrossheimin (**12**)	0.35 ± 0.18
Arglabin (**23**)	0.58 ± 0.11
Amphotericin B	0.14 ± 0.09

**Table 6:** Cytotoxicity of estafiatin (**1**), grossheimin (**4**), 8α-chloroacetoxygrossheimin (**12**), and arglabin (**23**) on RAW macrophages.

Compounds	CC_50_ (µg/mL) ± SD
Estafiatin (**1**)	<2
Grossheimin (**4**)	4.04 ± 1.40
8α-chloroacetoxygrossheimin (**12**)	<2
Arglabin (**23**)	2.50 ± 0.36

**Table 7:** Activity of estafiatin (**1**), grossheimin (**4**), and arglabin (**23**) against *Leishmania amazonensis* amastigotes.

Compounds	IC_50_ (µg/mL) ± SD	Selectivity Index
Estafiatin (**1**)	0.68 ± 0.007	<2.94
Grossheimin (**4**)	6.20 ± 2.46	<1
Arglabin (**23**)	1.13 ± 0.006	2.22
Amphotericin B	<0.924 (<0.004)	-

## Data Availability

The original contributions presented in this study are included in the article/Supplementary Materials. Further inquiries can be directed to the corresponding authors.

## Supplementary Materials

The following supporting information can be downloaded at: https://www.mdpi.com/article/10.3390/ijms27177971/s1.
