# Supplementary material for: Antiparasitic Activity of Sesquiterpene γ-Lactones and the Search for New Pharmaceutical Agents
Source: Int J Mol Sci. 2026 Sep 7;27(17):7971. doi: 10.3390/ijms27177971 (PMC13565976; doi:10.3390/ijms27177971)
Supplement: Supplementary file 1 [file ijms-27-07971-s001.zip › ijms-4202907-supplementary.pdf]

Supplementary material

Antiparasitic Activity of Sesquiterpene γ-Lactones and the Search for New Pharmaceutical Agents

Sergazy M. Adekenov [1,*], Laura C. Laurella [2,3], Shynggys Sergazy[1], Rachel Napoles Rodriguez [3], Augusto E. Bivona [4,5], Juan M. Viecenz [6], Orlando G. Elso [2,3], Aldana M. Corlatti [2], Nadia T. Mirakian [2], Esteban Bontempi [6], Paola A. Barroso [7], Dmitriy L. Savchenko [1], Balzhan Z. Medeubaeva [1], Gulimzhan S. Adekenova [1], Artur T. Boldysh [1], Anar N. Zhabayeva [1] and Valeria P. Sülsen [2,3,*]

1 JSC "Research Production Center "Phytochemistry", 100009, Karaganda, Kazakhstan; info@phyto.kz (S.M.A,); savchenkod_pharmatech@mail.ru (D.L.S.); medeubayeva_bz@mail.ru (B.Z.M.); g.uuuuu@bk.ru (G.S.A.); art.boldysh@gmail.com (A.T.B.); a.zhabaeva@phyto.kz (A.N.Z)
2 CONICET-Universidad de Buenos Aires, Instituto de Química y Metabolismo del Fármaco (IQUIMEFA), Buenos Aires, Argentina; c.laurella@docente.ffyb.uba.ar (L.C.L.); aldanamalencorlatti@gmail.com (A.M.C.); orlando.elso@gmail.com (O.G.E.); nadiatmirakian@gmail.com (N.T.M.)
3 Universidad de Buenos Aires, Facultad de Farmacia y Bioquímica, Cátedra de Farmacognosia, Buenos Aires, Argentina; rnapolesrodriguez@gmail.com (R.N.R)
4 Universidad de Buenos Aires, Facultad de Farmacia y Bioquímica, Cátedra de Inmunología, Buenos Aires, Argentina; abivona@docente.ffyb.uba.ar (A.E.B.)
5 CONICET – Universidad de Buenos Aires, Instituto de Estudios de la Inmunidad Humoral (IDEHU), Buenos Aires, Argentina; jmviecenz@hotmail.com.ar (J.M.V.); ejbon@yahoo.com (E.B.)
6 Instituto Nacional de Parasitología, ANLIS "Carlos G. Malbrán", Buenos Aires, Argentina
7 CONICET-Universidad Nacional de Salta, Instituto de Patología Experimental "Dr. Miguel Ángel Basombrío" (IPE), Salta A4400, Argentina; barrosopaola75@gmail.com (P.A.B.)
* Correspondence: arglabin@phyto.kz; Tel.: 8(7212) 43-31-27; vsulsen@ffyb.uba.ar (V.P.S.); Tel.: +54-(011)-5287-4272 (V.P.S.)

Figures

Figure S1. Dose-response curve of dihydroargolide (**3**) pulchellin C (**19**) and suramin against *T. b. brucei.*

Figure S2. Dose-response curve of dihydroargolide (3) pulchellin C (19) on *T. cruzi* amastigotes

Figure S3. Cytotoxicity of compounds 3 and 19 on Vero cells.

Figure S4. Dose-response curves of estafiatin (1), 8α-chloroacetoxygrossheimin (12), arglabin (23), and amphotericin B on *L. amazonensis* promastigotes.

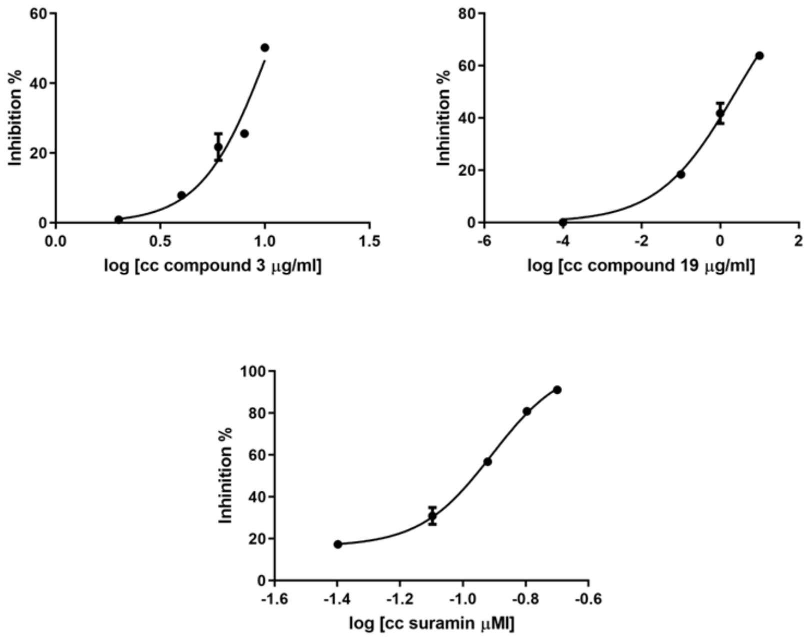

Figure S1. Dose-response curve of dihydroargolide (**3**) pulchellin C (**19**) and suramin against *T. b. brucei*.

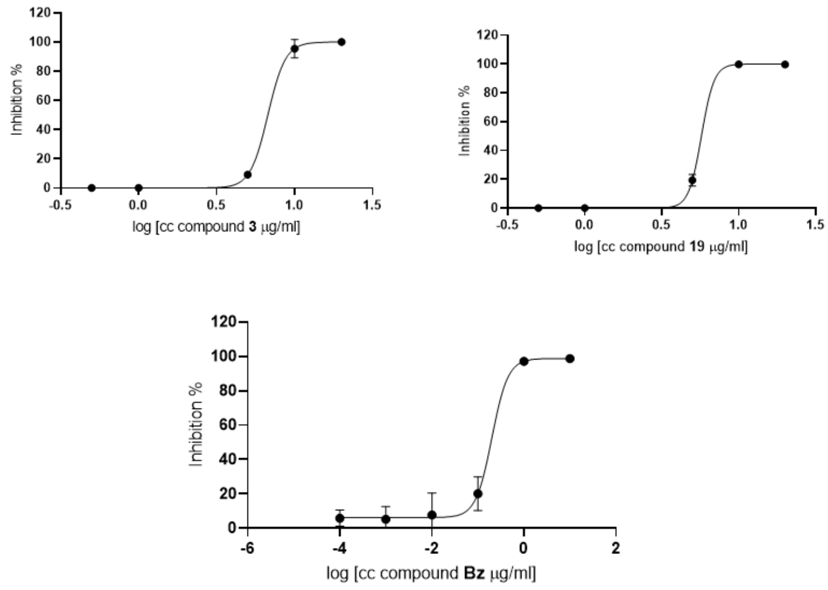

Figure S2. Dose-response curve of dihydroargolide (**3**) pulchellin C (**19**) on *T. cruzi* amastigotes. Bz: benznidazole

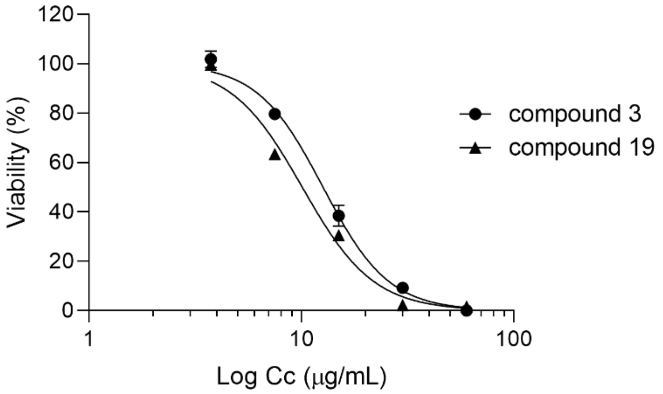

Figure S3. Cytotoxicity of compounds **3** and **19** on Vero cells.

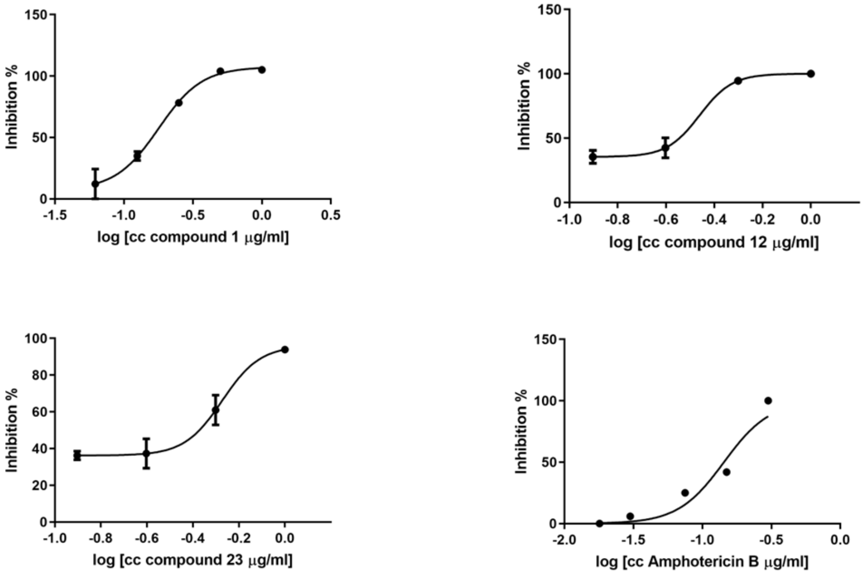

Figure S4. Dose-response curves of estafiatin (**1**), 8α-chloroacetoxygrossheimin (**12**), arglabin (**23**), and amphotericin B on *L. amazonensis* promastigotes.

Spectroscopic data for sesquiterpene lactones

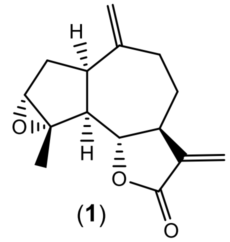

(**1**)

Estafiatin (**1**) – colorless needle-shaped crystals of composition $C_{15}H_{18}O_3$, m.p. 102-104 °C, $[\alpha]_D^{20}$ +312.50° (*c* 0.16, EtOH). The compound has the structure of 3α,4α-epoxy-1,5,7α(H),6β(H)-guai-10(14),11(13)-dien-6,12-olide.

IR spectrum (KBr, ν_{max}, cm^{-1}): 1753 (C=O of γ-lactone), 1685, 1653, 1637 (C=C).

UV spectrum (EtOH, λ_{max}, nm): 204.

PMR spectrum (500 MHz, $CDCl_3$, δ, ppm, J/Hz): 2.85-2.95 (2H, m, H-1, H-7), 1.89-1.91 (2H, m, H-2α, H-2β), 3.38 (1H, m, H-3), 2.16-2.30 (4H, m, H-5, H-8α, H-9α, H-9β), 4.20 (1H, dd, J = 11.01, 8.71 Hz, H-6), 1.48-1.58 (1H, m, H-8β), 4.90 (1H, d, J = 2.02 Hz, H-13α), 4.80 (1H, d, J = 2.02 Hz, H-13β), 6.09 (1H, d, J = 3.6 Hz, H-14α), 5.50 (1H, d, J = 3.6 Hz, H-14β), 1.50 (3H, s, CH_3-15).

^{13}C NMR spectrum (125.76 MHz, $CDCl_3$, δ, ppm): 45.27 (d, C-1), 32.75 (t, C-2), 63.57 (d, C-3), 65.96 (s, C-4), 50.89 (d, C-5), 80.83 (d, C-6), 43.60 (d, C-7), 28.84 (t, C-8), 27.86 (t, C-9), 140.32 (s, C-10), 146.74 (s, C-11), 170.44 (s, C-12), 114.10 (t, C-13), 119.18 (t, C-14), 17.55 (q, C-15).

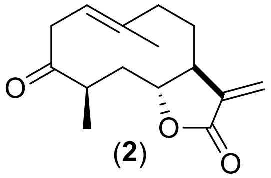

(**2**)

Argolide (**2**) – colorless crystalline substance of composition $C_{15}H_{20}O_3$, m.p. 133-135 °C (CH_2Cl_2-Et_2O), $[\alpha]_D^{20}$ +203.8° (*c* 0.52; $CHCl_3$). The compound has the structure of 3-keto-4α,7α(H),6β(H)-germacra-1(10)(E),11(13)-dien-6,12-olide.

IR spectrum (KBr, ν_{max}, cm^{-1}): 2999, 2966, 2928, 2870, 2850 (C-H), 1760 (C=O of γ-lactone), 1706 (C=O), 1663 (C=C), 1455, 1404, 1387, 1358, 1342, 1324, 1269, 1241, 1222, 1162, 1128, 1107, 1094, 1058, 1009, 971.

UV spectrum (EtOH, λ_{max}, nm): 206 ± 2.

Mass spectrum (70 eV, m/z, I_{rel}, %): M^+ 248 (9.28), 230 (2.14), 204 (10), 192 (5.71), 175 (5.71), 161 (2.85), 149 (100), 131 (9.28), 121 (34.28), 107 (11.42), 99 (22.85), 93 (62.85), 79 (37.14), 71 (14.28), 67 (25.71), 53 (40).

PMR spectrum (500 MHz, $CDCl_3$, δ, ppm, J/Hz): 1.12 (3H, d, J=6.7 Hz, CH_3-15); 1.32-1.24 (1H, m, H-5α); 1.60-1.54 (1H, m, H-8α); 1.64 (3H, s, CH_3-14); 1.99-1.88 (1H, m, H-8β); 2.08-2.12 (1H, m, H-5β); 2.40-2.30 (2H, m, H-9α, H-9β); 2.70 (1H, d, J=10.9 Hz, H-7); 2.90-2.80 (1H, m, H-4); 3.31-3.00 (2H, m, H-2α, H-2β); 3.66 (1H, dd, J=11.4, 2.4 Hz, H-6); 5.60-5.53 (1H, m, H-1); 5.67 (1H, d, J=1.2 Hz, H-13α); 6.24 (1H, d, J=1.6 Hz, H-13β).

^{13}C NMR spectrum (125.76 MHz, $CDCl_3$, δ, ppm): 117.15 (d, C-1); 42.60 (t, C-2); 208.12 (s, C-3); 39.95 (d, C-4); 39.86 (t, C-5); 81.82 (d, C-6); 43.90 (d, C-7); 34.89 (t, C-8); 40.24 (t, C-9); 141.59 (s, C-10); 140.33 (s, C-11); 170.03 (s, C-12); 123.21 (t, C-13); 15.89 (q, C-14); 18.48 (q, C-15).

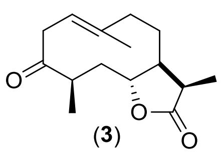

(**3**)

11,13-Dihydroargolide (**3**) – colorless crystalline substance of composition $C_{15}H_{22}O_3$, m.p. 170-171 °C (Et_2O-CH_2Cl_2), $[\alpha]_D^{22}$ +45.0° (*c* 0.43; $CHCl_3$). The compound has the structure of 3-keto-4,7α(H),6β,11β(H)-germacra-1(10)-en-6,12-olide.

IR spectrum (KBr, ν_{max}, cm^{-1}): 1765, 1710, 1675.

PMR spectrum (500 MHz, $CDCl_3$, δ, ppm, J/Hz): 1.05 (3H, d, J = 6.5 Hz, CH_3-15), 1.15 (3H, d, J = 6.5 Hz, CH_3-13), 1.59 (3H, br s, CH_3-14), 3.03 (2H, d, J = 8.6 Hz, H-2α, H-2β), 3.54 (1H, dd, J = 12, 10 Hz, H-6), 5.47 (1H, t, J = 8.5 Hz, H-1).

Mass spectrum (70 eV, m/z, I_{rel}, %): 250 [M^+] (28.76), 235 (10), 208 (12.85), 193 (55.71), 177 (55.71), 149 (14.28), 123 (47.14), 119 (28.57), 108 (100), 99 (82.85), 95 (62.85), 81 (58.57), 71 (35.71).

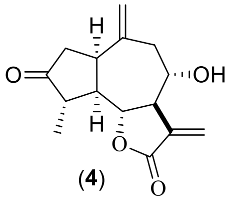

(**4**)

Grossheimin (**4**) – colorless crystalline substance of composition $C_{15}H_{18}O_4$, m.p. 200-202 °C (EtOH), $[\alpha]_D^{20}$ +159.9° (*c* 1.14; $CHCl_3$). The compound has the structure of 3-keto-8α-hydroxy-1α,5α,7α(H),4β,6β(H)-guai-10(14),11(13)-dien-6,12-olide.

IR spectrum (KBr, ν_{max}, cm^{-1}): 3475 (OH group), 3322, 3069, 2982, 2957, 2919, 2891, 1785 (C=O of γ-lactone), 1649 (C=O), 1443, 1399, 1357, 1293, 1260, 1240, 1181, 1073, 1040, 991, 956, 900.

UV spectrum (EtOH, λ_{max}, nm): 206 ± 2.

PMR spectrum (500 MHz, $CDCl_3$, δ, ppm, J/Hz): 1.18 (3H, d, J=6.73 Hz, CH_3-15); 2.19-2.31 (3H, m, H-4, H-9β, H-5); 2.42-2.55 (2H, m, H-2α, H-2β); 2.76-2.80 (1H, dd, J=18.62, 12.46 Hz, H-9α); 3.02-3.07 (1H, dd, J=12.46, 3.15 Hz, H-7); 3.12-3.16 (1H, m, H-1); 3.72-3.75 (1H, dd, J=9.31, 5.87 Hz, H-8); 3.95 (1H, dd, J=17.90, 9.02 Hz, H-6); 4.71 (1H, s, H-14α); 5.02 (1H, s, H-14β); 6.30 (2H, m, H-13α, H-13β).

^{13}C NMR spectrum (125.76 MHz, $CDCl_3$, δ, ppm): 40.26 (d, C-1); 43.29 (t, C-2); 219.10 (s, C-3); 48.01 (d, C-4); 51.23 (d, C-5); 82.39 (d, C-6); 49.30 (d, C-7); 73.26 (d, C-8); 47.08 (t, C-9); 143.29 (s, C-10); 136.52 (s, C-11); 169.99 (s, C-12); 125.84 (t, C-13); 115.76 (t, C-14); 15.01 (q, C-15).

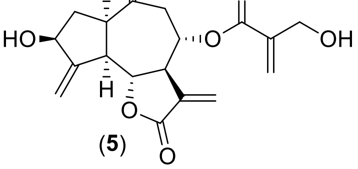

(**5**)

Cynaropicrin (**5**) – transparent yellowish oily substance of composition $C_{19}H_{22}O_6$; $[\alpha]_D^{20}$ +108.6° (*c* 1.19, EtOH). Decomposes on exposure to air with formation of a curdy precipitate, insoluble in $CHCl_3$ and

EtOAc. The compound has the structure of 3β-hydroxy-8α-[4′-hydroxymethacryloyl]-1α,5α,7α(H),6β,8β(H)-guai-4(15),10(14),11(13)-trien-6(12)-olide.

IR spectrum (KBr, ν_{max}, cm^{-1}): 3334 (OH), 1762 (C=O of γ-lactone), 1717 (C=O of methacryl) 1659, 1641 (C=C), 1405, 1150.

UV spectrum (EtOH, λ_{max}, nm): 204 (log ε 3.64).

PMR spectrum (500 MHz, $CDCl_3$, δ, ppm, J/Hz): 2.96 (1H, dd, J = 8.1, 7.5 Hz, H-1), 2.22 (1H, m, H-2α), 1.74 (1H, m, H-2β), 4.55 (1H, ttt, J = 7.2, 1.9, 1.9 Hz, H-3), 2.84 (1H, dd, J = 10.4, 9.0 Hz, H-5), 4.24 (1H, dd, J = 11.0, 9.1 Hz, H-6), 3.20-3.16 (1H, m, H-7), 5.14-5.13 (1H, m, H-8), 2.40 (1H, dd, J = 14.5, 3.8 Hz, H-9α), 2.72 (1H, dd, J = 14.5, 5.1 Hz, H-9β), 6.22 (1H, d, J = 3.4 Hz, H-13α), 5.61 (1H, d, J = 3.0 Hz, H-13β), 5.14 (1H, d, J = 0.9 Hz, H-14α), 4.93 (1H, d, J = 1.0 Hz, H-14β), 5.49 (1H, t, J = 1.8 Hz, H-15α), 5.36 (1H, t, J = 1.5 Hz, H-15β), 6.32 (1H, d, J = 1.2 Hz, H-3′α), 5.95 (1H, d, J = 0.9 Hz, H-3′β), 4.37 (2H, s, H-4′).

^{13}C NMR spectrum (125.76 MHz, $CDCl_3$, δ, ppm): 45.42 (d, C-1), 39.16 (t, C-2), 73.85 (d, C-3), 152.29 (s, C-4), 51.49 (d, C-5), 78.55 (d, C-6), 47.68 (d, C-7), 74.43 (d, C-8), 37.15 (t, C-9), 141.84 (s, C-10), 137.38 (s, C-11), 169.13 (s, C-12), 122.50 (t, C-13), 117.12 (t, C-14), 112.82 (t, C-15), 165.39 (s, C-1′), 139.36 (s, C-2′), 126.80 (t, C-3′), 60.50 (t, C-4′).

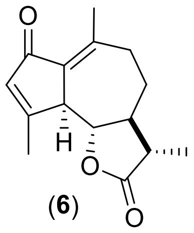

(**6**)

Leucomisin (**6**) – colorless crystalline substance of composition $C_{15}H_{18}O_3$, m.p. 196-198 °C (EtOH), $[\alpha]_D^{20}$ +56° (*c* 2.86, $CHCl_3$). The compound has the structure of 2-keto-5,7α(H),6β,11β(H)-guai-1(10),3(4)-dien-6,12-olide.

IR spectrum (KBr, ν_{max}, cm^{-1}): 2978, 2943, 2864, 1777 (C=O of γ-lactone), 1682, 1636, 1615 (dienone fragment), 1448, 1377, 1318, 1293, 1255, 1204, 1116, 1033, 986.

UV spectrum (EtOH, λ_{max}, nm): 256 (cyclopentadienone).

PMR spectrum (500 MHz, $CDCl_3$, δ, ppm, J/Hz): 1.24 (3H, d, J=6.9 Hz, CH_3-13); 1.38-1.31 (1H, m, H-8α); 1.94-1.90 (1H, m, H-8β); 2.00-1.95 (1H, m, H-9α); 2.24 (1H, dd, J=12.5, 6.9 Hz, H-11); 2.27 (3H, s, CH_3-15); 2.29 (1H, m, H-7); 2.38-2.37 (1H, m, H-9β); 2.41 (3H, s, CH_3-14); 3.39 (1H, d, J=10.2 Hz, H-5); 3.62 (1H, t, J=9.95 Hz, H-6); 6.14 (1H, br. s, H-3).

^{13}C NMR spectrum (125.76 MHz, $CDCl_3$, δ, ppm): 131.91 (s, C-1); 196.09 (s, C-2); 135.63 (d, C-3); 170.11 (s, C-4); 56.40 (d, C-5); 84.28 (d, C-6); 52.61 (d, C-7); 26.03 (t, C-8); 37.61 (t, C-9); 152.31 (s, C-10); 41.21 (d, C-11); 177.75 (s, C-12); 12.39 (q, C-13); 21.74 (q, C-14); 19.95 (q, C-15).

Mass spectrum (70 eV, m/z, I_{rel}, %): 247.1329 $[M+H]^+$ (calculated for $C_{15}H_{19}O_3$, 247.1329); MS/MS: 201.1276, 191.1435, 173.0962, 159.0799, 158.0725.

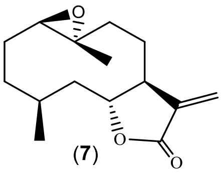

(**7**)

Epoxyargolide (**7**) – white crystalline substance with the molecular formula $C_{15}H_{20}O_4$, m.p. 203-205 °C (EtOAc). $[\alpha]_D^{23}$ +40.3° (*c* 1.19, $CHCl_3$). The compound has the structure of 1α,10β-epoxy-7α(H),4β,6β(H)-germacr-11(13)-en-6,12-olide.

IR spectrum (KBr, ν_{max}, cm^{-1}): 2982, 2969, 2932 (C-H), 2863, 1761 (C=O of γ-lactone), 1714 (C=O), 1662 (C=C), 1457, 1442, 1407, 1325, 1281, 1253, 1199, 1107, 1079, 1008 (C-O-C), 980, 949, 894, 818.

UV spectrum (EtOH, λ_{max}, nm): 208.

PMR spectrum (500 MHz, $CDCl_3$, δ, ppm, J/Hz): 3.31 (1H, dd, J = 9.9, 5.6 Hz; H-1), 3.26 (1H, dd, J = 14.4, 5.6 Hz; H-2α), 2.23-2.18 (2H, m, H-2β, H-5α), 2.97-2.90 (1H, m, H-4), 1.45 (1H, qd, J = 13.72, 12.53, 11.7, 3.8 Hz, H-5β), 4.05 (1H, dd, J = 11.7, 3.0 Hz; H-6), 2.78 (1H, d, J = 10.6 Hz; H-7), 1.70-1.65 (1H, m, H-8α), 1.98-1.89 (1H, m, H-8β), 2.28 (1H, td, J = 13.75, 3.65 Hz, H-9α), 1.27-1.25 (1H, m, H-9β), 5.70 (1H, d, J = 1.4 Hz; H-13α), 6.28 (1H, d, J = 1.0 Hz; H-13β), 1.28 (3H, s, CH_3-14), 1.16 (3H, d, J = 6.7 Hz; CH_3-15).

^{13}C NMR spectrum (125.76 MHz, $CDCl_3$, δ, ppm): 59.02 (d, C-1), 44.03 (t, C-2), 210.23 (s, C-3), 40.02 (d, C-4), 39.89 (t, C-5), 80.95 (d, C-6), 44.15 (d, C-7), 33.79 (t, C-8), 39.52 (t, C-9), 59.08 (s, C-10), 140.18 (s, C-11), 169.29 (s, C-12), 123.79 (t, C-13), 18.24 (q, C-14), 15.67 (q, C-15).

Elemental analysis: Found (%): C 67.96; H 7.25; O 24.79. Calculated (%): C 68.16; H 7.63; O 24.21.

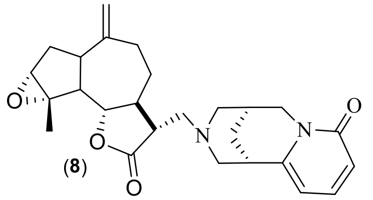

(**8**)

Cytisinyl-estafiatin (**8**) – colorless crystalline substance with the molecular formula $C_{26}H_{32}N_2O_4$, m.p. 214-217 °C. The compound has the structure of 3α,4α-epoxy-13α-cytisinyl-7α(H),6β(H)-guai-10(14)-en-6,12-olide.

IR spectrum (KBr, ν_{max}, cm^{-1}): 2973, 2967, 2945, 2934, 2799 (C-H), 1757 (C=O of γ-lactone), 1652 (C=N), 1591, 1577, 1568 (C=C), 1462, 1450, 1378, 1329, 1300, 1209 (epoxide ring), 1191 (C-N), 1075, 1048 (CH_2), 985, 957, 826, 802 (C-O-C).

UV spectrum (EtOH, λ_{max}, nm): 203.

PMR spectrum (500 MHz, $CDCl_3$, δ, ppm, J/Hz): 1.93 (1H, m, H-1), 1.79 (1H, m, H-2α), 1.96 (1H, m, H-2β), 3.29 (1H, s, H-3), 1.86 (1H, m, H-5), 3.81 (1H, t, J = 10.0 Hz, H-6), 1.93 (1H, m, H-7), 2.20 (1H, ddd, J = 20.0, 9.7, 2.5 Hz, H-8α), 2.79 (1H, dd, J = 9.7, 5.7 Hz, H-8β), 1.68 (1H, ddd, J = 13.96, 10.52, 6.0 Hz, H-9α), 2.00 (1H, dd, J = 13.96, 7.3 Hz, H-9β), 2.10 (1H, dd, J = 11.0, 8.5 Hz, H-11), 2.47 (1H, dd, J = 13.5, 10.0 Hz, H-13α), 2.72 (1H, dd, J = 13.5, 3.5 Hz, H-13β), 4.74 (1H, s, H-14α), 4.79 (1H, s, H-14β), 1.50 (3H, s, CH_3-15), 6.44 (1H, dd, J = 9.0, 1.1 Hz, H-3′), 7.25 (1H, dd, J = 9.0, 6.8 Hz, H-4′), 5.96 (1H, dd, J = 6.8, 1.1 Hz, H-5′), 2.95 (1H, br s, H-7′), 1.74 (1H, m, H-8′α), 1.88 (1H, m, H-8′β), 2.42 (1H, br s, H-9′), 3.87 (1H, dd, J = 15.0, 10.0 Hz, H-10′α), 4.02 (1H, d, J = 15.0 Hz, H-10′β), 2.49 (1H, br d, J = 11.0 Hz, H-11′α), 2.86 (1H, br d, J = 11.0 Hz, H-11′β), 2.25 (1H, dd, J = 11.0, 2.0 Hz, H-13′α), 2.88 (1H, br d, J = 11.0 Hz, H-13′β).

^{13}C NMR spectrum (125.76 MHz, $CDCl_3$, δ, ppm): 30.59 (d, C-1), 31.12 (t, C-2), 63.27 (d, C-3), 66.04 (s, C-4), 48.01 (d, C-5), 80.81 (d, C-6), 44.40 (d, C-7), 44.54 (t, C-8), 32.77 (t, C-9), 146.84 (s, C-10), 50.40 (d, C-11), 177.04 (s, C-12), 58.58 (t, C-13), 114.35 (t, C-14), 18.79 (q, C-15), 163.35 (s, C-2′), 116.95 (d, C-3′), 138.47 (d, C-4′), 104.56 (d, C-5′), 151.31 (s, C-6′), 35.25 (d, C-7′), 25.78 (t, C-8′), 28.24 (d, C-9′), 50.01 (t, C-10′), 62.47 (t, C-11′), 59.46 (t, C-13′).

Elemental analysis: Found (%): C 71.58; H 7.42; N 6.36. Calculated (%): C 71.56; H 7.34; N 6.42.

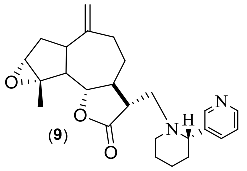

(**9**)

Anabasinyl-estafiatin (**9**) – colorless crystalline substance with the molecular formula $C_{25}H_{32}N_2O_3$, m.p. 131.8-134.6 °C (EtOAc:Hexane). The compound has the structure of 3α,4α-epoxy-13α-anabasinyl-7α(H),6β(H)-guai-10(14)-en-6,12-olide.

IR spectrum (KBr, ν_{max}, cm^{-1}): 2984, 2947, 2932, 2912 (C-H), 1774 (C=O of γ-lactone), 1633 (C=N), 1591, 1577, 1568 (C=C), 1453, 1443, 1427, 1327, 1303, 1209 (epoxide ring), 1199 (C-N), 1054, 1024, 1003 (CH_2), 985, 961, 826, 802 (C-O-C).

UV spectrum (EtOH, λ_{max}, nm): 203.

PMR spectrum (500 MHz, $CDCl_3$, δ, ppm, J/Hz): 3.10 (1H, m, H-1), 2.18 (2H, m, H-2α, H-2β), 3.34 (1H, s, H-3), 2.30 (1H, m, H-5), 3.95 (1H, t, J = 10.5 Hz, H-6), 2.35 (1H, m, H-7), 2.12 (2H, m, H-8α, H-8β), 2.22 (2H, m, H-9α, H-9β), 1.55 (1H, m, H-11), 2.31 (1H, dd, J = 13.53, 2.72 Hz, H-13α), 2.66 (1H, dd, J = 13.53, 6.87 Hz, H-13β), 4.87 (1H, s, H-14α), 4.92 (1H, s, H-14β), 1.53 (3H, s, CH_3-15), 1.69 (1H, m, H-2′), 1.72 (2H, m, H-3′α, H-3′β), 1.78 (1H, d, J = 1.8 Hz, H-4′α), 2.30 (1H, d, J = 1.8 Hz, H-4′β), 1.33 (2H, m, H-5′α, H-5′β), 2.84 (1H, d, J = 10.5 Hz, H-6′α), 2.93 (1H, d, J = 10.0 Hz, H-6′β), 8.50 (1H, d, J = 1.8 Hz, H-8′), 7.66 (1H, d, J = 7.8 Hz, H-10′), 7.25 (1H, dd, J = 7.8, 1.8 Hz, H-11′), 8.48 (1H, dd, J = 4.8, 1.8 Hz, H-12′).

^{13}C NMR spectrum (125.76 MHz, $CDCl_3$, δ, ppm): 30.21 (d, C-1), 30.67 (t, C-2), 63.47 (d, C-3), 66.33 (s, C-4), 44.75 (d, C-5), 80.79 (d, C-6), 42.64 (d, C-7), 44.75 (t, C-8), 32.88 (t, C-9), 147.12 (s, C-10), 51.05 (d, C-11), 178.05 (s, C-12), 54.05 (t, C-13), 114.37 (t, C-14), 18.66 (q, C-15), 66.12 (d, C-2′), 36.46 (t, C-3′), 24.56 (t, C-4′), 25.72 (t, C-5′), 51.92 (t, C-6′), 148.76 (d, C-8′), 140.30 (s, C-9′), 135.56 (d, C-10′), 123.65 (d, C-11′), 149.61 (d, C-12′).

Elemental analysis: Found (%): C 73.51; H 7.86; N 6.85. Calculated (%): C 73.53; H 7.84; N 6.86.

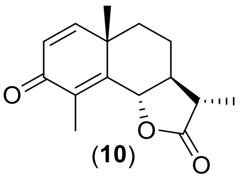

(**10**)

α-Santonin (**10**) – colorless crystalline substance of composition $C_{15}H_{18}O_3$, m.p. 169-171 °C, $[\alpha]_D^{20}$ -171.8° (*c* 0.2; CH_3OH). The compound has the structure of 3-keto-6,11β,7α(H)-eudesma-1(2),4(5)-dien-6,12-olide.

IR spectrum (KBr, ν_{max}, cm^{-1}): 2974, 2936, 2870 (C-H), 1785 (C=O of γ-lactone), 1658 (C=O), 1629, 1612 (C=C), 1457, 1406, 1376, 1267, 1244, 1193, 1179, 1153, 1135, 1103, 1033, 991.

UV spectrum (EtOH, λ_{max}, nm): 240 (log ε 3.77).

PMR spectrum (500 MHz, $CDCl_3$, δ, ppm, J/Hz): 1.19 (3H, d, J = 6.87 Hz, CH_3-13); 1.26 (3H, s, CH_3-14); 1.44 (1H, ddd, J = 13.75, 13.46, 5.15 Hz, H-9α); 1.65 (1H, dq, J = 12.6, 12.03, 3.44 Hz, H-8α); 1.75 (1H, dq, J = 12.03, 11.46, 3.44 Hz, H-7); 1.85 (1H, dq, J = 13.46, 3.44, 2.29 Hz, H-9β); 1.99-1.94 (1H, m, H-8β); 2.04 (3H, s, CH_3-15); 2.38 (1H, q, J = 5.15 Hz, H-10); 4.76 (1H, dd, J = 10.88, 1.15 Hz, H-6); 6.16 (1H, d, J = 9.74 Hz, H-2); 6.64 (1H, d, J = 9.74 Hz, H-1).

^{13}C NMR spectrum (125.76 MHz, $CDCl_3$, δ, ppm): 155.03 (d, C-1); 125.83 (d, C-2); 186.39 (s, C-3); 128.56 (s, C-4); 151.31 (s, C-5); 81.45 (d, C-6); 53.59 (d, C-7); 22.91 (t, C-8); 37.87 (t, C-9); 41.00 (s, C-10); 41.48 (d, C-11); 177.79 (s, C-12); 12.57 (q, C-13); 25.18 (q, C-14); 10.98 (q, C-15).

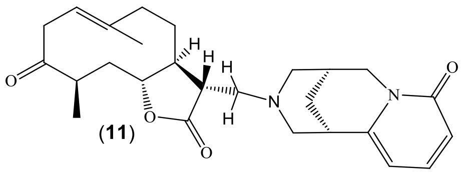

(**11**)

13α-Cytisinylargolide (**11**) – colorless crystalline substance with m.p. 185-187 ˚C, of composition $C_{26}H_{34}O_4N_2$. The compound has the structure of 3-keto-13α-cytisinyl-4α,7α(H),6β,11β(H)-germacr-1(10)-en-6,12-olide.

IR spectrum (KBr, ν_{max}, cm^{-1}): 3030, 2994, 2925, 2884, 2852, 2796, 2360, 2341, 1766 (C=O of γ-lactone), 1705, 1658, 1581, 1569, 1547, 1472, 1449, 1424, 1373, 1360, 1336, 1311, 1238, 1215, 1202, 1175, 1065, 980, 802, 711, 675, 558.

UV spectrum (EtOH, λ_{max}, nm): 203, 234, 311.

PMR spectrum (500 MHz, $CDCl_3$, δ, ppm, J/Hz): 5.29 (1H, t, J = 8.5 Hz, H-1), 2.98 (3H, m, H-7′, H-2α, H-2β), 2.66 (1H, m, H-4), 1.40 (1H, ddd, J = 14.2, 9.9, 3.3 Hz, H-5α), 1.94 (4H, m, H-9β, H-8′β, H-7, H-5β), 3.59 (1H, d, J = 9.0 Hz, H-6), 1.56 (1H, td, J = 14.2, 13.0, 3.9 Hz, H-8a), 1.09 (1H, dt, J = 14.3, 3.4 Hz, H-8β), 1.75 (2H, m, H-8′α, H-9α), 2.25 (1H, ddd, J = 11.6, 5.2, 4.0 Hz, H-11), 2.44 (1H, dd, J = 12.7, 11.7 Hz, H-13α), 2.69 (1H, dd, J = 12.9, 5.2 Hz, H-13β), 1.48 (3H, s, CH_3-14), 1.03 (3H, d, J = 6.7 Hz, CH_3-15), 6.46 (1H, dd, J = 9.0, 1.4 Hz, H-3′), 7.30 (1H, dd, J = 9.0, 6.9 Hz, H-4′), 6.00 (1H, dd, J = 6.9, 1.3 Hz, H-5′), 2.51 (1H, m, H-9′), 3.94 (1H, dd, J = 15.3, 6.6 Hz, H-10′α), 4.02 (1H, d, J = 15.5 Hz, H-10′β), 2.57 (1H, d, J = 11.0 Hz, H-11′α), 2.82 (1H, m, H-11′β), 2.29 (1H, d, J = 10.8 Hz, H-13′α), 2.89 (1H, m, H-13′β).

^{13}C NMR spectrum (125.76 MHz, $CDCl_3$, δ, ppm): 118.03 (d, C-1), 42.23 (t, C-2), 208.95 (s, C-3), 39.86 (d, C-4), 39.04 (t, C-5), 84.27 (d, C-6), 40.40 (d, C-7), 33.03 (t, C-8), 39.63 (t, C-9), 140.50 (s, C-10), 47.07 (d, C-11), 177.77 (s, C-12), 58.86 (t, C-13), 15.89 (q, C-14), 18.33 (q, C-15), 163.40 (s, C-2′), 117.07 (d, C-3′), 139.01 (d, C-4′), 104.36 (d, C-5′), 151.51 (s, C-6′), 35.14 (d, C-7′), 25.69 (t, C-8′), 28.18 (d, C-9′), 50.31 (t, C-10′), 62.62 (t, C-11′), 58.72 (t, C-13′).

Elemental analysis: Found (%): C 71.02; H 7.74; N 6.37. Calculated (%): C 71.23; H 7.76; N 6.39.

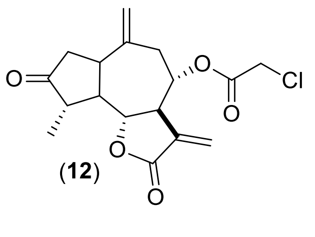

(**12**)

8α-Chloroacetoxygrossheimin (**12**) – white powdery substance with m.p. 162-164 ˚C (EtOH), of composition $C_{17}H_{19}O_5Cl$. The compound has the structure of 3-keto-8α-chloroacetoxy-5α,7α(H),4β,6β,8β(H)-guai-10(14),11(13)-dien-6,12-olide.

IR spectrum (KBr, ν_{max}, cm^{-1}): 1754 (C=O of γ-lactone), 1723 (C=O), 1647 (C=C), 1453, 1417, 1360, 1338, 1316, 1296, 1278, 1240 (C=O of ester), 1207, 1143, 821 (C-Cl).

UV spectrum (EtOH, λ_{max}, nm): 205 (ε 1.225), 258 (ε 0.5585).

PMR spectrum (500 MHz, $CDCl_3$, δ, ppm, J/Hz): 6.36 (1H, d, J = 3.3 Hz, H-13α), 5.92 (1H, d, J = 2.9 Hz, H-13β), 5.15 (1H, s, H-14α), 5.01 (1H, m, H-14β), 4.88 (2H, m, H-17α, H-17β), 4.14 (1H, d, J = 0.8 Hz, H-8), 4.06 (1H, d, J = 9.1 Hz, H-6), 3.38 (1H, m, H-7), 3.16 (1H, m, H-1), 3.00 (1H, dd, J = 13.0, 5.8 Hz, H-5), 2.56 (1H, dd, J = 19.1, 8.7 Hz, H-9β), 2.49 (1H, dd, J = 19.2, 3.4 Hz, H-4), 2.37-2.24 (3H, m, H-2α, H-2β, H-9α), 1.25 (3H, d, J = 6.7 Hz, 15-Me).

^{13}C NMR spectrum (125.76 MHz, $CDCl_3$, δ, ppm): 43.08 (d, C-1), 43.23 (t, C-2), 218.62 (s, C-3), 46.21 (d, C-4), 51.00 (d, C-5), 82.15 (d, C-6), 45.58 (d, C-7), 76.33 (d, C-8), 40.11 (t, C-9), 141.75 (s, C-10), 135.26 (s, C-11), 169.37 (s, C-12), 125.51 (t, C-13), 117.43 (t, C-14), 14.94 (q, C-15), 166.26 (s, C-16), 40.78 (t, C-17).

Elemental analysis: Found (%): C 59.39; H 5.41; Cl 12.15. Calculated (%): C 60.26; H 5.6; Cl 10.5.

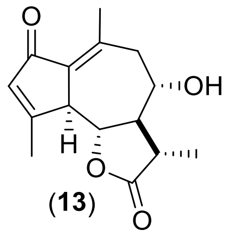

(**13**)

Austricin (**13**) – colorless crystalline substance of composition $C_{15}H_{18}O_4 \cdot H_2O$, m.p. 149-151 °C, $[\alpha]_D^{20}$ +13.52° (*c* 3.7; EtOH). The compound has the structure of 2-keto-8α-hydroxy-5α,7α(H),6β,8β(H)-guai-3(4),1(10)-dien-6,12-olide.

IR spectrum (KBr, ν_{max}, cm^{-1}): 3550 (OH group), 1775 (C=O of γ-lactone), 1685, 1645, 1630 cm^{-1} (cyclopentadienone).

UV spectrum (EtOH, λ_{max}, nm): 255 (ε 14425).

PMR spectrum (500 MHz, $CDCl_3$, δ, ppm, J/Hz): 1.44 (3H, d, J=7.0 Hz, CH_3-13); 2.12 (1H, dt, J=11.5, 10.0 Hz, H-7); 2.25-2.21 (1H, m, H-9β); 2.41 (3H, s, CH_3-14); 2.51-2.58 (1H, m, H-9α); 2.78 (1H, dd, J=11.0, 7.4 Hz, H-11); 2.88 (3H, s, CH_3-15); 3.37 (1H, d, J=10.0 Hz, H-5); 3.64 (1H, t, J=10.6 Hz, H-8); 3.73 (1H, t, J=10.1 Hz, H-6); 6.15 (1H, s, H-3).

^{13}C NMR spectrum (125.76 MHz, $CDCl_3$, δ, ppm): 133.0 (s, C-1); 195.7 (s, C-2); 135.7 (d, C-3); 170.3 (s, C-4); 61.5 (d, C-5); 81.1 (d, C-6); 69.6 (d, C-7); 41.4 (d, C-8); 51.7 (t, C-9); 145.8 (s, C-10); 49.1 (d, C-11); 177.8 (s, C-12); 15.6 (q, C-13); 20.0 (q, C-14); 21.8 (q, C-15).

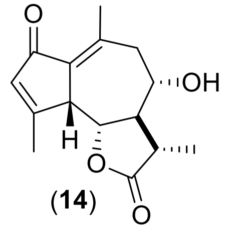

(**14**)

5β(H)-Austricin (**14**) – colorless crystalline substance of composition $C_{15}H_{18}O_4$, m.p. 258-260 °C (EtOAc), $[\alpha]_D^{25}$ +147.06° (*c* 0.034; $CHCl_3$). The compound has the structure of 2-keto-8α-hydroxy-7α(H),5β,6β,8β,11β(H)-guai-1(10),3(4)-dien-6,12-olide.

IR spectrum (KBr, ν_{max}, cm^{-1}): 3287, 1794, 1665, 1628, 1611, 1435, 1381, 1318, 1162, 1004, 954, 884, 865, 631, 590.

UV spectrum (EtOH, λ_{max}, nm): 255 (cyclopentadienone).

PMR spectrum (500 MHz, $CDCl_3$, δ, ppm, J/Hz): 1.40 (3H, d, J = 7.0 Hz, CH_3-13), 1.97 (1H, ddd, J = 12.0, 10.5, 7.0 Hz, H-7), 2.13 (3H, s, CH_3-15), 2.27 (3H, s, CH_3-14), 2.47 (1H, dd, J = 19.0, 10.0 Hz, H-9α), 2.60 (1H, dq, J = 12.0, 7.0 Hz, H-11), 3.27 (1H, dd, J = 19.0, 6.0 Hz, H-9β), 3.45 (1H, br s, J = 4.0 Hz, H-5), 4.20 (1H, dd, J = 7.0, 4.0 Hz, H-6), 4.58 (1H, ddd, J = 10.5, 10.0, 6.0 Hz, H-8), 6.12 (1H, m, H-3).

^{13}C NMR spectrum (125.76 MHz, $CDCl_3$, δ, ppm): 128.41 (s, C-1), 196.46 (s, C-2), 135.61 (d, C-3), 168.16 (s, C-4), 55.81 (d, C-5), 73.95 (d, C-6), 51.64 (d, C-7), 73.05 (d, C-8), 42.75 (t, C-9),145.16 (s, C-10), 45.44 (d, C-11), 177.65 (s, C-12), 14.62 (q, C-13), 16.64 (q, C-14), 19.70 (q, C-15).

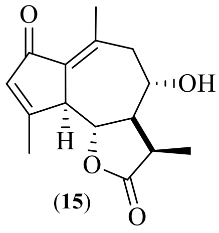

(**15**)

Grossmisin (**15**) – yellowish powdery substance of composition $C_{15}H_{18}O_4$, m.p. 166-167 °C, $[\alpha]_D^{20}$ +115.26° (*c* 1.2; EtOH). The compound has the structure of 2-keto-8α-hydroxy-5α,7α,11α(H),6β,8β(H)-guai-1(10),3(4)-dien-6,12-olide.

IR spectrum (KBr, ν_{max}, cm^{-1}): 3503 (OH), 3322, 3069, 2982, 2957, 2919, 2891 (C-H), 1785 (C=O of γ-lactone), 1753, 1680, 1636, 1608 (dienone fragment), 1446, 1380, 1293, 1261, 1222, 1197, 1062, 1032, 977, 939.

UV spectrum (EtOH, λ_{max}, nm): 255 (cyclopentadienone).

PMR spectrum (500 MHz, $CDCl_3$, δ, ppm, J/Hz): 1.29 (3H, d, J=7.66 Hz, CH_3-13); 2.30 (3H, s, CH_3-15); 2.44 (3H, s, CH_3-14); 2.48 (1H, dd, J=2.0, 13.5 Hz, H-9α); 2.55 (1H, dd, J=17.65, 10.4 Hz, H-7); 2.75 (1H, t, J=12.14 Hz, H-9β); 2.94 (1H, q, J=14.89 Hz, H-11); 3.41 (1H, d, J=10.10 Hz, H-5); 3.84 (1H, t, J=10.3 Hz, H-6); 3.72 (1H, t, J=9.7 Hz, H-8); 6.18 (1H, s, H-3).

^{13}C NMR spectrum (125.76 MHz, $CDCl_3$, δ, ppm): 132.71 (s, C-1); 196.12 (s, C-2); 135.46 (d, C-3);170.97 (s, C-4); 51.89 (d, C-5); 81.10 (d, C-6); 58.00 (d, C-7); 64.96 (d, C-8); 48.24 (t, C-9); 146.81 (s, C-10); 38.16 (d, C-11);178.77 (s, C-12); 9.45 (q, C-13); 21.83 (q, C-14); 19.91 (q, C-15).

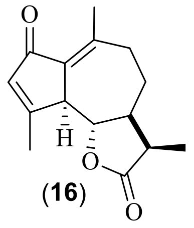

(**16**)

Achillin (**16**) – colorless crystalline substance of composition $C_{15}H_{18}O_3$ with m.p. 144-145.5 °C, $[\alpha]_D^{24}$ +160° (*c* 1.20; $CHCl_3$). The compound has the structure of 2-keto-5α,7α,11α(H),6β(H)-guai-1(10),3(4)-dien-6,12-olide.

IR spectrum (KBr, ν_{max}, cm^{-1}): 2967, 2935, 2918, 2894, 2879, 2864 (C-H), 1777 (C=O of γ-lactone), 1680, 1617, 1633 (dienone fragment), 1461, 1435, 1386, 1363, 1322, 1259, 1213, 1199, 1166, 1154, 1133, 1044, 983.

UV spectrum (EtOH, λ_{max}, nm): 256 ± 2 (cyclopentadienone).

PMR spectrum (500 MHz, $CDCl_3$, δ, ppm, J/Hz): 1.22 (3H, d, J=7.73 Hz, CH_3-13); 1.41 (1H, dd, J=12.5, 1.58 Hz, H-8α); 1.87-1.81 (1H, m, H-8β); 2.41 (3H, s, CH_3-14); 2.27 (3H, s, CH_3-15); 2.31 (1H, dtd, J=14.5, 6.0, 1.72 Hz, H-9α); 2.41-2.40 (1H, m, H-9β); 2.47-2.42 (1H, m, H-7); 2.69 (1H, q, J=7.73 Hz, H-11); 3.40 (1H, d, J=9.88 Hz, H-5); 3.79 (1H, t, J=10.31 Hz, H-6); 6.15 (1H, br. s, H-3).

^{13}C NMR spectrum (125.76 MHz, $CDCl_3$, δ, ppm): 131.84 (s, C-1); 196.06 (s, C-2); 135.63 (d, C-3); 170.21 (s, C-4); 53.02 (d, C-5); 83.58 (d, C-6); 52.05 (d, C-7); 23.68 (t, C-8); 37.69 (t, C-9); 152.29 (s, C-10); 39.45 (d, C-11); 178.60 (s, C-12); 10.04 (q, C-13); 21.65 (q, C-14); 19.91 (q, C-15).

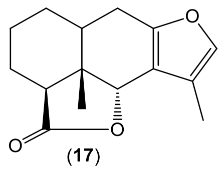

(**17**)

Furanoeremophilan-14β,6α-olide (**17**) – white crystalline substance of composition $C_{15}H_{18}O_3$, m.p. 135.7-137.1 ˚C (Pet. ether-EtOAc).

IR spectrum (KBr, ν_{max}, cm^{-1}): 3143, 3103 (C-H of furan ring), 3000, 2954, 2938, 2884, 2874, 1772 (C=O of γ-lactone), 1681 and 1639 (C=C), 1580, 1565, 1469, 1452, 1424, 1367, 1332, 1304, 1238, 1216, 1186, 1148, 1137, 1080, 1041, 1006, 970, 944, 867, 841, 831, 792, 776, 764, 729.

UV spectrum (EtOH, λ_{max}, nm): 216 nm.

PMR spectrum (500 MHz, $CDCl_3$, δ, ppm, J/Hz): 1.26 (3H, s, CH_3-13); 1.59-1.45 (3H, m, H-1α, H-2α, H-3α); 1.94-1.85 (2H, m, H-1β, H-3β); 1.81-1.75 (1H, m, H-2β); 2.02 (3H, s, CH_3-15); 2.32 (1H, dd, J=12.0, 3.0 Hz, H-4); 5.09 (1H, s, H-6); 2.70-2.60 (2H, m, H-9α, H-9β); 2.28-2.22 (1H, m, H-10); 7.25 (1H, s, H-12);

^{13}C NMR spectrum (125.76 MHz, $CDCl_3$, δ, ppm): 18.86 (t, C-1); 20.60 (t, C-2); 25.35 (t, C-3); 41.43 (d, C-4); 41.56 (s, C-5); 81.80 (d, C-6); 120.21 (s, C-7); 150.92 (s, C-8); 23.26 (t, C-9); 37.07 (d, C-10); 114.78 (s, C-11); 138.70 (d, C-12); 8.39 (q, C-13); 176.90 (s, C-14) ; 20.16 (q, C-15).

Elemental analysis: Found (%): C 73.06; H 7.39. Calculated (%): C 73.15; H 7.37.

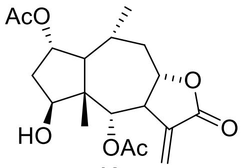

(**18**)

Britannin (**18**) – crystalline substance of composition $C_{19}H_{26}O_7$, m.p. 188-190 ˚C (EtOH), $[\alpha]_D^{20}$ -26˚ (*c* 5.0; $CHCl_3$). The compound has the structure of 2α,6α-diacetoxy-4β-hydroxy-6β,8β,10β(H)-pseudoguai-11(13)-en-7,12-olide.

IR spectrum (KBr, ν_{max}, cm^{-1}): 3540 (hydroxyl group), 1768 (C=O of γ-lactone), 1710, 1728 (C=O), 1240-1270 (O-C-O), 1668 (C=C).

UV spectrum (EtOH, λ_{max}, nm): 254.

Mass spectrum (70 eV, m/z, I_{rel}, %): 246 $[M\text{-}2\times HOAc]^+$, 228 $[M\text{-}2\times HOAc\text{-}H_2O]^+$.

PMR spectrum (500 MHz, $CDCl_3$, δ, ppm, J/Hz): 6.14 (1H, dd, J = 2, 1.5 Hz, H-13α), 5.36 (1H, d, J = 3.2 Hz, H-13β), 5.04 (1H, d, J = 8.5 Hz, H-6), 4.87 (1H, dd, J = 6, 9 Hz, H-2), 4.50 (1H, dd, J = 10, 11.5 Hz, H-8), 4.11 (1H, dd, J = 9, 10.5 Hz, H-4), 3.01 (1H, m, H-7), 2.41 (2H, m, H-9α, 4-OH), 2.22 (3H, s, 2-$OCOCH_3$), 2.02 (3H, s, 6-$OCOCH_3$), 2.05 (1H, m, H-3α), 1.89 (2H, m, H-1, H-10), 1.79 (1H, dd, J = 8.5, 15 Hz, H-3β), 1.42 (1H, dt, J = 11.7, 13.3 Hz, H-9β), 1.00 (3H, s, CH_3-15), 0.96 (3H, d, J = 5 Hz, CH_3-14).

^{13}C NMR spectrum (125.76 MHz, $CDCl_3$, δ, ppm): 51.0 (d, C-1), 75.2 (d, C-2), 36.2 (t, C-3), 72.8 (d, C-4), 50.9 (s, C-5), 76.3 (d, C-6), 52.2 (d, C-7), 76.1 (d, C-8), 43.9 (t, C-9), 30.0 (d, C-10), 138.4 (s, C-11), 168.8 (s, C-12), 120.0 (t, C-13), 15.8 (q, C-14), 20.3 (q, C-15), 170.2 (s, 2-$O\underline{C}OCH_3$), 172.4 (s, 6-$O\underline{C}OCH_3$), 21.0 (q, 2-$OCO\underline{C}H_3$), 21.1 (q, 6-$OCO\underline{C}H_3$).

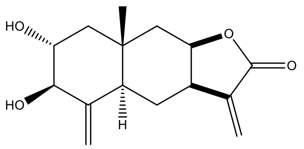

(**19**)

Pulchellin C (**19**) – colorless crystalline substance of composition $C_{15}H_{20}O_4$, M^+ 264, m.p. 200-203 °C, $[\alpha]_D^{20}$ +92.8° (*c* 0.0045; $CHCl_3$). The compound has the structure of 2α,3β-dihydroxy-5,7,8α(H)-eudesma-4(15),11(13)-dien-7,12-olide.

IR spectrum (KBr, ν_{max}, cm^{-1}): 3600, 3490 (-OH); 1760 (C=O of γ-lactone); and 1660 (double bond).

PMR spectrum (500 MHz, $CDCl_3$, δ, ppm, J/Hz): 0.83 (3H, s, CH_3-14); 2.24 (1H, dd, J = 15, 1.5 Hz, H-5); 2.73 (2H, br s, 2-OH, 3-OH); 2.94 (1H, q; J = 6 Hz, H-7); 3.54 (1H, m, H-2), 3.83 (1H, d, J = 9 Hz, H-3), 4.56 (1H, m, H-8); 4.72 (1H, s, H-15α), 5.23 (1H, s, H-15β); 5.62 (1H, d, J = 1 Hz, H-13α), 6.15 (1H, d, J = 1 Hz, H-13β).

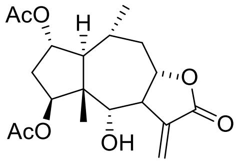

(**20**)

Inuchinenolide C (**20**) – colorless crystalline substance of composition $C_{19}H_{26}O_7$, m.p. 156-158 °C, $[\alpha]_D^{20}$ +95.5° (*c* 0.0045; $CHCl_3$). The compound has the structure of 2α,4β-diacetoxy-6α-hydroxy-1α(H),8β,10β(H)-pseudoguai-11(13)-en-7,12-olide.

IR spectrum (KBr, ν_{max}, cm^{-1}): 3540 (OH); 1780 (C=O of γ-lactone); 1760, 1740 (acetyl group C=O); 1680.

Mass spectrum (70 eV, m/z, I_{rel}, %): 246 $[M-2\times HOAc]^+$, 228 $[M-2\times HOAc-H_2O]^+$.

PMR spectrum (500 MHz, $CDCl_3$, δ, ppm, J/Hz): 6.20 (1H, d, J = 3.5 Hz, H-13α), 5.98 (1H, d, J = 3 Hz, H-13β), 5.61 (1H, dd, J = 8.5, 9 Hz, H-4), 4.94 (1H, br t, J = 8 Hz, H-2), 4.87 (1H, br s, 6-OH), 4.45 (1H, m, H-8), 3.52 (1H, d, J = 6.5 Hz, H-6), 2.14 (3H, s, 2-$OCOCH_3$), 2.04 (3H, s, 4-$OCOCH_3$), 0.99 (3H, d, J = 6 Hz, 14-CH_3), 0.91 (3H, s, 15-CH_3).

^{13}C NMR spectrum (125.76 MHz, $CDCl_3$, δ, ppm): 50.0 (d, C-1), 76.2 (d, C-2), 44.2 (t, C-3), 76.0 (d, C-4), 52.3 (s, C-5), 75.0 (d, C-6), 53.9 (d, C-7), 74.5 (d, C-8), 35.3 (t, C-9), 30.4 (d, C-10), 139.5 (s, C-11), 170.0 (s, C-12), 121.7 (t, C-13), 17.3 (q, C-14), 20.8 (q, C-15), 173.2 (s, 2-$O\underline{C}OCH_3$), 170.2 (s, 4-$O\underline{C}OCH_3$), 21.3 (q, 2-$OCO\underline{C}H_3$), 21.4 (q, 4-$OCO\underline{C}H_3$).

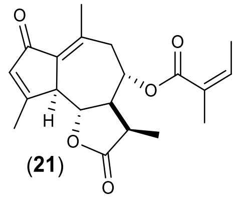

(**21**)

B

adkhysin (**21**) – white crystalline substance of composition $C_{20}H_{24}O_5$, m.p. 139-140 °C. The compound has the structure of 2-keto-8α-angeloyl-5α,7α,11α(H),6β(H)-guai-1(10),3(4)-dien-6,12-olide.

IR spectrum (KBr, ν_{max}, cm^{-1}): 2973, 2963, 2923, 2909, 2890 (C-H), 1769 (C=O of γ-lactone), 1708, 1695, 1684, 1652, 1641, 1617 (dienone moiety), 1459, 1433, 1340, 1321, 1300, 1237, 1215, 1183, 1157, 1131, 1077, 1036, 1020, 977, 898, 851, 844.

UV spectrum (EtOH, λ_{max}, nm): 253 ± 2.

PMR spectrum (500 MHz, CD_3OD, δ, ppm, J/Hz): 1.33 (3H, s, CH_3-5′); 1.87 (3H, d, J=3.0 Hz, CH_3-4′); 1.99 (3H, d, J=7.50 Hz, CH_3-13); 2.24 (3H, s, CH_3-15); 2.25 (1H, dd, J=18.40, 9.45 Hz, H-7); 2.27 (3H, s, CH_3-14); 2.95-2.81 (2H, m, H-9α, H-9β); 3.08-3.04 (1H, m, H-11); 3.58 (1H, d, J=10.67, H-5); 4.45 (1H, dd, J=10.67, 8.23 Hz, H-8); 5.49 (1H, ddd, J=10.04, 9.74, 3.72 Hz, H-6); 6.15 (1H, s, H-3); 6.18 (1H, dd, J=7.5, 1.5 Hz, H-3′).

^{13}C NMR spectrum (125.76 MHz, $CDCl_3$, δ, ppm): 129.84 (s, C-1); 195.38 (s, C-2); 135.48 (d, C-3); 166.68 (s, C-4); 49.12 (d, C-5); 60.84 (d, C-6); 45.17 (d, C-7); 67.13 (d, C-8); 43.62 (t, C-9); 145.36 (s, C-10); 37.30 (d, C-11); 178.19 (s, C-12); 13.38 (q, C-13); 16.02 (q, C-14); 19.80 (q, C-15); 169.82 (s, C-1′); 127.04 (s, C-2′); 140.21 (d, C-3′); 20.61 (q, C-4′); 20.32 (q, C-5′).

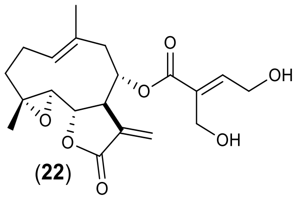

(**22**)

Stizolicin (**22**) – colorless crystalline substance of composition $C_{20}H_{26}O_7$, m.p. 152-154 °C, $[\alpha]_D^{20}$ -32.4° (*c* 2.19; EtOH). The compound has the structure of 4α,5α-epoxy-8α-[(E)-4′-hydroxy-2′-(hydroxymethyl)but-2′-enoate]-7α(H),6β(H)-germacra-1(10),11(13)-dien-6,12-olide.

IR spectrum (KBr, ν_{max}, cm^{-1}): 3479, 3452 (OH), 2981, 2967, 2950, 2939, 2920, 2897, 2883, 2862 (C-H), 1765 (C=O of γ-lactone), 1717, 1663, 1651 (dienone fragment), 1472, 1462, 1439, 1394, 1383, 1366, 1330, 1284, 1239, 1194, 1157, 1072, 1031, 1024, 950.

UV spectrum (EtOH, λ_{max}, nm): 203 (log ε = 4.25).

PMR spectrum (500 MHz, $CDCl_3$, δ, ppm, J/Hz): 1.28 (3H, s, CH_3-15); 1.30-1.27 (1H, m, H-3α); 1.81 (3H, s, CH_3-14); 2.14-1.98 (1H, m, H-3β); 2.2-2.28 (1H, m, H-2α); 2.54-2.42 (2H, m, H-2β, H-9α); 2.69 (1H, d, J=12.03 Hz, H-9β); 2.83 (1H, d, J=9.45 Hz, H-5); 3.57 (1H, m, H-7); 4.26 (1H, d, J=9.00 Hz, H-6); 4.40-4.34 (4H, m, H-4′α, H-4′β, H-5′α, H-5′β); 4.58-4.51 (1H, m, H-8); 5.43 (1H, d, J=12.03 Hz, H-1); 5.78 (1H, d, J=2.94 Hz, H-13α); 6.18 (1H, d, J=3.44 Hz, H-13β); 6.93 (1H, t, J=6.00 Hz, H-3′).

^{13}C NMR spectrum (125.76 MHz, $CDCl_3$, δ, ppm): 129.66 (d, C-1); 23.92 (t, C-2); 35.60 (t, C-3); 61.18 (s, C-4); 66.19 (d, C-5); 80.92 (d, C-6); 46.38 (d, C-7); 73.69 (d, C-8); 49.02 (t, C-9); 131.17 (s, C-10); 133.90 (s, C-11); 170.20 (s, C-12); 127.16 (t, C-13); 17.05 (q, C-14); 16.27 (q, C-15); 166.49 (s, C-1′); 124.74 (s, C-2′); 145.69 (d, C-3′); 55.33 (t, C-4′); 58.06 (t, C-5′).

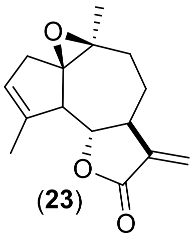

(**23**)

Arglabin (**23**) – colorless crystals of composition $C_{15}H_{18}O_3$; m.p. 100-102 °C (hexane), $[\alpha]_D^{20}$ +45.6° (*c* 0.3; $CHCl_3$). The compound has the structure of 1,10β-epoxy-5,7α,6,11β(H)-guai-3,4-en-6,12-olide.

IR spectrum (KBr, ν_{max}, cm^{-1}): 2999, 2940, 2925, 2851 (C-H), 1765 (C=O of γ-lactone), 1665 (C=C), 1432, 1408, 1379, 1333, 1307, 1281, 1254, 1222, 1199, 1155, 1135, 1111, 1091, 1063 (epoxide ring).

UV spectrum (EtOH, λ_{max}, nm): 204.

PMR spectrum (500 MHz, $CDCl_3$, δ, ppm, J/Hz): 1.33 (3H, s, CH_3-14); 1.52-1.43 (1H, m, H-8β); 1.83 (1H, dd, J=5.01, 2.51 Hz, H-8α); 1.95 (3H, s, CH_3-15); 2.04-1.97 (1H, m, H-9β); 2.14-2.10 (1H, m, H-2β); 2.21-2.15 (1H, m, H-9α); 2.27-2.23 (1H, m, H-7); 2.79-2.73 (1H, m, H-2α); 2.95-2.90 (1H, dq, J=11.20, 1.07 Hz, H-5); 3.98 (1H, dd, J=10.74, 9.74 Hz, H-6); 5.40 (1H, d, J=3.37 Hz, H-13α); 5.55 (1H, ddd, J=4.50, 2.65, 1.72 Hz, H-3); 6.12 (1H, d, J=3.37 Hz, H-13β).

^{13}C NMR spectrum (125.76 MHz, $CDCl_3$, δ, ppm): 62.77 (s, C-1); 21.49 (t, C-2); 124.98 (d, C-3); 140.57 (s, C-4); 52.86 (d, C-5); 82.96 (d, C-6); 51.06 (d, C-7); 33.49 (t, C-8); 39.75 (t, C-9); 72.58 (s, C-10); 139.15 (s, C-11); 170.55 (s, C-12); 118.39 (t, C-13); 22.84 (q, C-14); 18.31 (q, C-15).

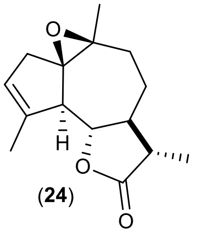

(**24**)

Arborescin (**24**) – colorless crystalline substance of composition $C_{15}H_{20}O_3$, m.p. 139-142 °C, $[\alpha]_D^{19}$ +70.1° ($CHCl_3$). The compound has the structure of 1β,10β-epoxy-5α,7α(H),6β,11β(H)-guai-3(4)-en-6,12-olide.

IR spectrum (KBr, ν_{max}, cm^{-1}): 3056, 3008, 2967, 2948, 2926, 2868, 2852, 2831 (C-H), 1766 (C=O of γ-lactone), 1654 (C=C), 1456, 1444, 1379, 1367, 1348, 1285, 1244, 1223, 1187, 1178, 1126, 1113, 1102, 1065, 1029 (epoxy cycle).

UV spectrum (EtOH, λ_{max}, nm): 204 (ε 18521).

PMR spectrum (500 MHz, $CDCl_3$, δ, ppm, J/Hz): 1.18 (3H, d, J=6.9 Hz, CH_3-13); 1.33 (3H, s, CH_3-14); 1.45 (1H, m, H-8β); 1.64 (1H, m, H-8α); 1.76 (1H, m, H-7); 1.93 (3H, br.s, CH_3-15); 2.08 (1H, m, H-9β); 2.14 (1H, m, H-2β); 2.18 (1H, m, H-9α); 2.76 (1H, m, H-2α); 2.95 (1H, d, J=10.0 Hz, H-5); 3.15 (1H, dq, J=12.3 Hz, 6.9, H-11); 4.02 (1H, t, J = 10.0 Hz, H-6); 5.55 (1H, s, H-3).

^{13}C NMR spectrum (125.76 MHz, $CDCl_3$, δ, ppm): 76.0 (s, C-1), 43.3 (t, C-2), 128.4 (d, C-3), 144.3 (s, C-4), 58.1 (d, C-5), 86.2 (d, C-6), 55.9 (d, C-7), 22.0 (t, C-8), 37.1 (t, C-9), 66.1 (s, C-10), 44.5 (d, C-11), 182.2 (s, C-12), 16.1 (q, C-13), 26.4 (q, C-14), 26.5 (q, C-15).
